# Co‐activation pattern alterations in autism spectrum disorder–A volume‐wise hierarchical clustering fMRI study

**DOI:** 10.1002/brb3.2174

**Published:** 2021-05-16

**Authors:** Jyri‐Johan Paakki, Jukka S. Rahko, Aija Kotila, Marja‐Leena Mattila, Helena Miettunen, Tuula M. Hurtig, Katja K. Jussila, Sanna Kuusikko‐Gauffin, Irma K. Moilanen, Osmo Tervonen, Vesa J. Kiviniemi

**Affiliations:** ^1^ Faculty of Medicine, Health and Biosciences Doctoral Programme University of Oulu Graduate School University of Oulu Oulu Finland; ^2^ The Faculty of Medicine Research Unit of Medical Imaging, Physics and Technology Oulu Functional NeuroImaging Group University of Oulu Oulu Finland; ^3^ Department of Diagnostic Radiology Medical Research Center Oulu University Hospital Oulu Finland; ^4^ PEDEGO Research Unit Faculty of Medicine Child Psychiatry University of Oulu Oulu Finland; ^5^ Institute of Clinical Medicine Clinic of Child Psychiatry University and University Hospital of Oulu Oulu Finland; ^6^ Faculty of Humanities Research Unit of Logopedics University of Oulu Oulu Finland; ^7^ Research Unit of Clinical Neuroscience, Psychiatry University of Oulu Oulu Finland

**Keywords:** adolescent, autism spectrum disorder, brain, CAP, fMRI, hierarchical clustering, resting state network

## Abstract

**Introduction:**

There has been a growing effort to characterize the time‐varying functional connectivity of resting state (RS) fMRI brain networks (RSNs). Although voxel‐wise connectivity studies have examined different sliding window lengths, nonsequential volume‐wise approaches have been less common.

**Methods:**

Inspired by earlier co‐activation pattern (CAP) studies, we applied hierarchical clustering (HC) to classify the image volumes of the RS‐fMRI data on 28 adolescents with autism spectrum disorder (ASD) and their 27 typically developing (TD) controls. We compared the distribution of the ASD and TD groups' volumes in CAPs as well as their voxel‐wise means. For simplification purposes, we conducted a group independent component analysis to extract 14 major RSNs. The RSNs' average *z*‐scores enabled us to meaningfully regroup the RSNs and estimate the percentage of voxels within each RSN for which there was a significant group difference. These results were jointly interpreted to find global group‐specific patterns.

**Results:**

We found similar brain state proportions in 58 CAPs (clustering interval from 2 to 30). However, in many CAPs, the voxel‐wise means differed significantly within a matrix of 14 RSNs. The rest‐activated default mode‐positive and default mode‐negative brain state properties vary considerably in both groups over time. This division was seen clearly when the volumes were partitioned into two CAPs and then further examined along the HC dendrogram of the diversifying brain CAPs. The ASD group network activations followed a more heterogeneous distribution and some networks maintained higher baselines; throughout the brain deactivation state, the ASD participants had reduced deactivation in 12/14 networks. During default mode‐negative CAPs, the ASD group showed simultaneous visual network and either dorsal attention or default mode network overactivation.

**Conclusion:**

Nonsequential volume gathering into CAPs and the comparison of voxel‐wise signal changes provide a complementary perspective to connectivity and an alternative to sliding window analysis.

## Graphical abstract

Co‐activation patterns (CAPs) of resting state brain activity varyingly contain both default mode network (DMN)‐positive and default mode network‐negative brain state properties. With hierarchical clustering, they can be divided into shorter substates. When applied in autism spectrum disorder, this method showed simultaneous visual network and either dorsal attention or DMN overactivation during DMN‐negative CAPs. Nonsequential volume gathering into CAPs provides an alternative to sliding window analysis.

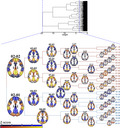

## 1 INTRODUCTION

According to the latest draft of WHO's International Classification of Diseases, autism spectrum disorder (ASD) is usually a pervasive but contextually varying feature of an individual's functioning observed in all settings: for example, personal, family, social, educational, and occupational interactions. ASD is characterized by persistent deficits in one's ability to initiate and sustain reciprocal social interaction and social communication, as well as by a range of restricted, repetitive, and inflexible patterns of behavior and interests (World Health Organization, 2018). Subjects with ASD prefer to focus on only a restricted number of repeating and controllable sensory information instead of the subtle and irregular cues of multisensory information needed in social communication. The etiology and expression of ASD are highly diverse, and the recent changes in the diagnostic criteria from categorical to dimensional reflect this growing understanding (Betancur, 2011; Lord & Jones, 2012; Mattila et al., 2011; Park et al., 2016; Varghese et al., 2017; Waye & Cheng, 2017).

Resting state (RS) functional magnetic resonance imaging (fMRI) examines spontaneous brain function by using blood oxygen level‐dependent (BOLD) contrast in the absence of a task. Traditionally, RS‐fMRI analysis has relied on a temporally stationary functional connectivity (FC) measure, in which the correlations between the voxel time series of brain regions are examined as unchanging over time. Previously, ASD has been affiliated with altered RS intrinsic FC, and the literature supports a diffuse pattern of both, rather than only under‐ or overconnectivity. Even a recent, thorough review of RS FC in ASD with nearly 70 analysis citations found it challenging to draw direct conclusions about the nature of FC in ASD (Hull et al., 2017). Since the seminal work of Chang and Glover (2010), the last decade has seen a growing trend and a deliberate effort to characterize dynamic changes in brain connectivity as a function of time, dynamic FC (dFC) or time‐varying FC (TVFC). The most widely used temporal sliding window approaches to between‐voxel correlations have demonstrated that FC in the brain has time‐varying properties (Allen et al., 2014; Chang & Glover, 2010; Hutchison et al., 2013; Kiviniemi et al., 2011; Lurie et al., 2020; Preti et al., 2017). It has been suggested that greater intraindividual dynamic variance is a potential biomarker of not only ASD but also mental disorders such as schizophrenia and attention deficit hyperactivity disorder and that it may underlie confusing static FC measures (Chen et al., 2017; Falahpour et al., 2016; Zhang et al., 2016). It is also important to recognize that the same static FC pattern could result from many different combinations or sequences of shorter spatiotemporal patterns of underlying TVFC (Lurie et al., 2020).

Of the existing TVFC methods, the co‐activation pattern (CAP) approach deviates from conventional time‐domain approaches by regarding single fMRI volumes at individual time points, instead of fMRI time courses, as the basic units of analysis (Liu et al., 2018). Hindriks et al. (2016) suggest that CAPs could be the building blocks of spontaneous BOLD activity and that dFC is a reflection of these. As Tagliazucchi et al. (2016) state: "Instead of asking whether two voxels are engaged in synchronized fluctuations over a relatively long period of time, the question is shifted to whether two voxels become jointly activated (i.e., present high activity above their baseline levels) and what are the timings and properties of these co‐activations." The beginning of the development of CAP analysis can be traced to when Tagliazucchi et al. (2012) established their point process analysis and observed that the timing of high‐activity events in BOLD signals allows the reconstruction of major RS networks (RSNs). Soon after, Liu and Duyn (2013) applied K‐means clustering to arrange single fMRI volumes into groups and averaged this data group‐wise to produce distinct spatial CAPs. Related studies have been conducted by other groups using CAP (Bolton et al., 2020; Chen et al., 2015; Di Perri et al., 2017; Zhuang et al., 2018; recent CAP review by Liu et al., 2018) or other terms such as coincident threshold crossings (Hudetz et al., 2015) and modes (Li et al., 2015).

**FIGURE 1:**
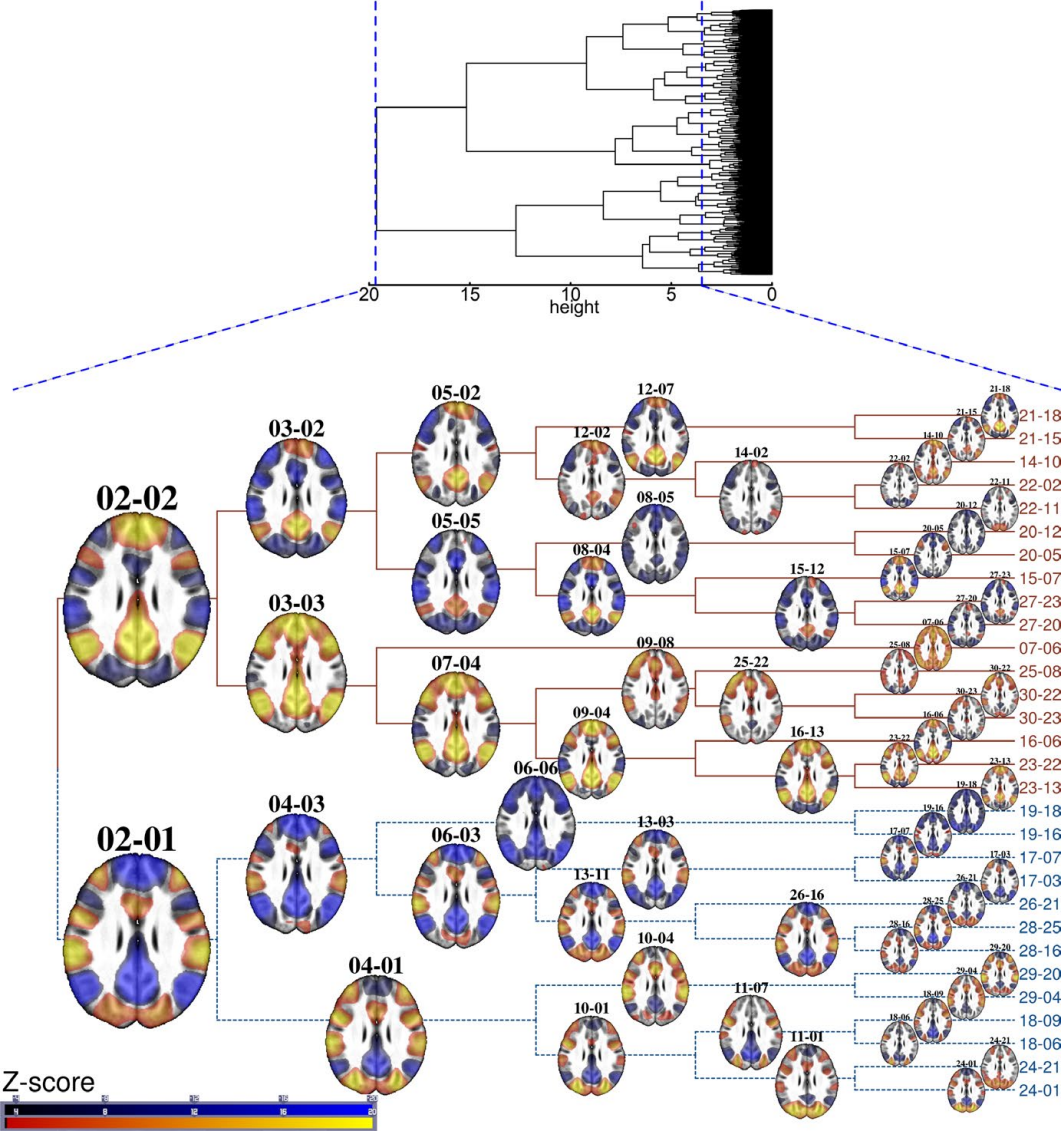
Hierarchical clustering result dendrogram. Top, hierarchical clustering (cosine distance, Ward's method) result dendrogram of 11,930 resting state (RS) fMRI volumes of 55 adolescent participants. The *x*‐axis shows the study‐specific distances between the clusters as height h. When h = 0, each volume forms its own cluster, and h≈3.48 (dashed blue line on the right) corresponds to splitting the volumes into 30 clusters. Due to image resolution limitations, the volumes were merged as the thickened black column on the *y*‐axis. The lower part of the figure highlights the cluster levels from 02 to 30 as a cladogram, which shows the relations between the CAPs. The first number indicates the total cluster count at that level. The second number was determined by the clustering algorithm that showed the cluster's ordinal number for only that hierarchical level. For the sake of visualization, in the cladogram, the branch lengths have been scaled equal, as opposed to a dendrogram. Each CAP's *z*‐statistic map slice is shown from the same level of anterior and dorsal nodes of the default mode network (DMN). The lower DMN‐negative or "task‐positive" branches of the cladogram comprise 4,658 volumes (39%), and the upper DMN‐positive branches 7,272 volumes (61% of the whole data) of the RS‐fMRI data. Further CAP volume distributions (Figures S1a‐b) are detailed in Appendix S1. The *z*‐statistic color keys range from −20 to −3.5 and from 3.5 to 20

**FIGURE 2:**
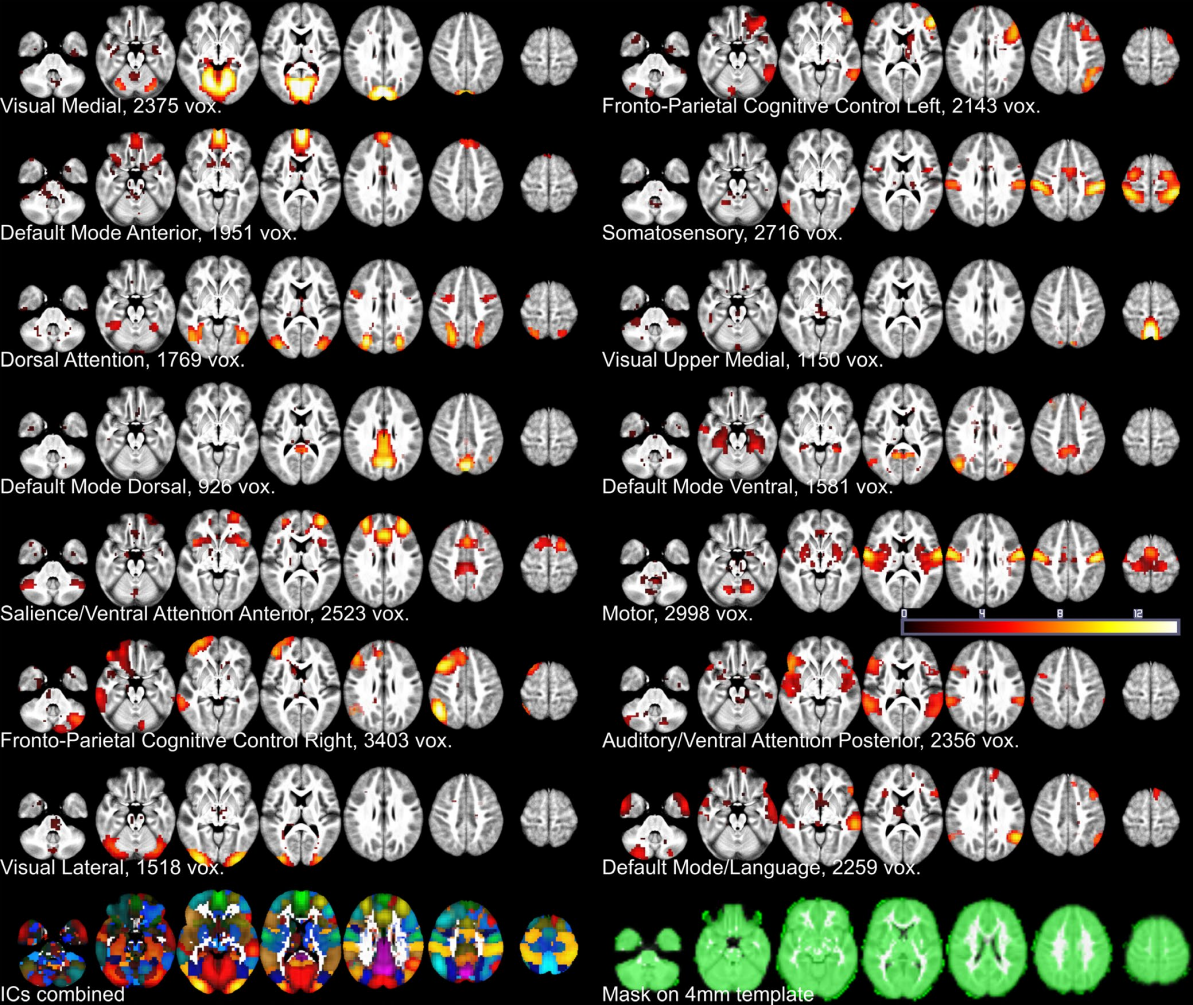
Study‐specific cores of 14 resting state networks (FSL MELODIC group ICA components). The components are ordered column‐wise according to the explained variance. Overlapping areas were removed by assigning each voxel to only one IC, where it had the highest absolute *z*‐statistic over the other ICs. The component areas were only used as voxel‐wise masks and a simple atlas to label brain areas when interpreting CAP results. Images of the combined 14 components and study mask are also displayed. The *z*‐statistic color key ranges from 0 (black) to 14 (white)

Constellations of different intrinsic connectivity network (ICN) patterns have previously been referred to as brain states (Allen et al., 2014). Although in our study, CAPs are not calculated as time‐varying FC matrix representations as illustrated by Allen et al. (2014), they can be seen as representing the same brain states, as a timeline of various spatiotemporal clusters of (de)activation patterns of independent RSNs (Preti et al. 2017). These brain states are visualized as a mean image of multiple volumes (Figure 1) in which RSNs (Figure 2) are in sufficiently similar phases of activation as determined by a clustering algorithm. If the number of states or CAPs depicting the same data is increased, the amount of time for which one CAP is represented grows proportionately shorter. In CAP analysis, the gathered volumes do not have to be sequential, and voxel signal levels per se can be evaluated. These two issues present the major differences from most sliding window TVFC analyses (Liu et al., 2018; Lurie et al., 2020; Preti et al., 2017).

From the CAP methods, the following can be distinguished 1) seed‐based methods (e.g., Amico et al., 2014; Chen et al., 2015; Di Perri et al., 2017; Liu & Duyn, 2013), in which the volumes for analysis are selected via predefined seed voxel or region thresholds and in which interactions with the rest of the brain are probed, and 2) seed‐free analyses, such as ours, in which the clustering algorithm is applied to all volumes in an entirely data‐driven way (e.g., Bolton et al., 2020; Liu et al., 2013, 2018). Another relevant distinction between studies is that most earlier studies have focused on temporal properties or CAP metrics, such as the occurrence rate, dwell time, and transition probability (e.g., Bolton et al., 2020; Chen et al., 2015), whereas the most salient findings in our study concentrate on the spatial differences across the groups (also see Amico et al., 2014; Di Perri et al., 2017).

In CAP analysis, BOLD fMRI volumes are described by their voxels' signal amplitudes, and their relation to other volumes can be defined via a suitable function, such as the Pearson correlation coefficient (Chen et al., 2015; Di Perri et al., 2017; Li et al., 2015; Liu & Duyn, 2013; Yao et al., 2016) or cosine distance (Karahanoğlu & Van De Ville, 2015). fMRI studies have utilized hierarchical clustering methods with a voxel‐wise approach (Liu et al., 2012; Thirion et al., 2014; Wang & Li, 2013; Wang et al., 2016) and with FC analysis (Kam et al., 2017). Agglomerative algorithms are deterministic and do not require a predetermined number of clusters (as opposed to k‐means clustering). To decide which data objects (here fMRI volumes) are combined during hierarchical clustering, we chose Ward's method. This belongs to minimum variance methods and uses one variant of the Lance–Williams dissimilarity update formula, sharing the total error sum of squares criterion with k‐means clustering (Murtagh & Legendre, 2014).

As individual CAPs reflect the continuous flow of time‐varying information within functional brain networks (Liu et al., 2018), we wanted to study whether the effects of restricted, narrow focusing of attention to limited information sources typical to ASD can be detected in the clustering of CAP information. To determine possible CAP differences between adolescents with ASD and typically developing (TD) controls, we compared the distribution of the ASD and TD groups' volumes in CAPs as well as their voxel‐wise means. This enabled us to determine the specific differences between the (de)activation patterns of the two groups. The number of CAPs in earlier studies ranges from 4 (Li et al., 2015) to 30 (Liu et al., 2013), and we addressed this range accordingly.

## 2 MATERIALS AND METHODS

### 2.1 Dataset

The study population, detailed diagnostic criteria, and all the data were the same as those used by Paakki et al. (2010). As fMRI preprocessing methods have advanced, in the present study, we utilized them as described below.

The study population consisted of 55 adolescents, of which 28 had ASD (with normal IQ, age 14.58 ± 1.62 years, 20 males, 8 females, three left‐handed) according to ICD‐10 research criteria (World Health Organization, 1993) and 27 were TD‐matched controls (age 14.49 ± 1.51 years, 18 males, 9 females, two left‐handed). The MR data were collected using a GE 1.5 T Signa HDX scanner with an eight‐channel parallel imaging head coil. Before the scan, the participants were asked to lie still, remain relaxed and awake, and look at a white cross in the middle of a dark‐gray screen. The 7.5 min RS BOLD fMRI scanning consisted of 253 whole brain volumes. The parameters of the gradient‐recalled echo‐planar imaging (GRE EPI) were as follows: TR 1.8 s, TE 40 ms, flip angle 90°, FOV 256 mm, 64 × 64 in‐plane matrix, 4 × 4 × 4 mm voxel size, and 28 oblique axial slices with a 0.4 mm gap and interleaved acquisition order. We acquired the structural data using a T1‐weighted 3D FSPGR sequence with 1 mm oblique axial slices, FOV 24.0 × 24.0 cm with a 256 × 256 matrix.

All participants and their parents gave written informed consent. The study was approved by the Regional Ethics Committee of the Northern Ostrobothnia Hospital District and conducted in accordance with the Declaration of Helsinki.

### 2.2 Preprocessing of RS‐fMRI signals

For anatomical data, we used FSL‐VBM FNIRT to register individual T1 structural head volumes and generate a study‐specific template (Andersson et al., 2007; Douaud et al., 2007; Good et al., 2001; Smith et al., 2004). After skull stripping, the FSL's FIRST segmented cerebrospinal fluid (CSF), white matter (WM), and gray matter (GM) (Patenaude et al., 2011).

For the functional data, AFNI's (Cox, 1996; Cox & Hyde, 1997; Gold et al., 1998) "afni_proc.py" program produced preprocessing pipeline according to the program's help page examples 9b and 10 (https://afni.nimh.nih.gov/pub/dist/doc/program_help/afni_proc.py.html) and following the guidelines of Jo et al. (2013). We removed the first three volumes to avoid T1 effects and computed the outlier fractions for each volume. Motion correction was applied, and the skull was stripped. Exploiting the results of our earlier FSL‐VBM procedure and the study‐specific average template, we applied a nonlinear transformation to functional data.

After these steps, we interrupted the AFNI pipeline and ran the independent component (IC) analysis‐based automatic removal of motion artifacts (ICA‐AROMA) for the functional data. The median number of ICs left after artifact removal was 14.5 in ASD and 13 in the TD group (Wilcoxon rank‐sum test *p*‐value = .41). The nonaggressive option was implemented in the participant‐wise removal of artifact components (Pruim et al., 2015; Pruim, Mennes, van Rooij, et al., 2015).

The AFNI pipeline continued with despiking. The volumes with a displacement of >0.2 mm or with a normalized signal level of over 1.29 (outlying 10% of expected *SD*) were labeled for censoring (Aurich et al., 2015; Nichols, 2013; Power et al., 2014, 2015). On average, 13.2% (min. 0%, max. 45.6%) of the time series were censored. The shortest time left was 4 min 5 s, which was still considered adequate (White et al., 2014). After censoring, 13,750 volumes were reduced to 11,930: The TD participants had an average of 223 volumes, and the ASD participants 210 volumes left (Wilcoxon rank‐sum *p*‐value = .34).

For full details on preprocessing, Appendix S1. We did not regress the global signal. We performed spatial smoothing with 8 mm (~2 voxels) full width at half maximum kernel (Chen & Calhoun, 2018) and calculated high pass temporal filtering regressors for frequencies of <.005 Hz. Removal of trends, censoring, temporal filtering, motion, once eroded CSF mask, and local WM (ANATICOR) regressors were combined into a regression matrix with AFNI 3dDeconvolve and projected out of the smoothed data in one step with the AFNI 3dTproject to remove any possible residual noise (Jo et al., 2010, 2013).

### 2.3 Group independent component analysis

We created uncensored but otherwise similarly preprocessed datasets with FSL MELODIC multisession temporal concatenation analysis and estimated group‐level ICs. These were used as masks and a simple atlas to label brain areas when interpreting the CAP results. We adjusted the dimensionality to 14 ICs and chose the low dimensional approach for the sake of pragmatic visual pattern analysis, but still covered major networks in line with earlier studies (Castellazzi et al., 2014; Smith et al., 2009; Starck et al., 2013; Thornburgh et al., 2017; Yeo et al., 2011, 2015).

### 2.4 Hierarchical clustering and extraction of CAPs

The preprocessing continued in MATLAB^®^ (MathWorks^®^, 2016; Shen, 2014). The fMRI signal was temporally normalized voxel‐wise for each participant by subtracting the mean and then dividing by the temporal *SD* (Liu et al., 2018). The individual datasets were masked using combined GICA components and GM voxels (Figure 2). These volumes and mask were later used with FSL randomise.

The volumes were reshaped and concatenated, and the resulting data matrix was transferred to the R environment (Bengtsson, 2016; R Core Team, 2017). We applied clustering to all the BOLD fMRI volumes acquired from the 55 participants that had survived censoring. As mentioned in the introduction, the volumes are described by their voxels' signal amplitudes, and their relation to other volumes has to be defined via a suitable function. Here, individual volumes were represented as 29,684‐dimensional vectors, and a matrix containing the pairwise cosine similarity among all the 11,930 vectors was calculated.

As we were interested in the spatial similarity of the volumes and the corresponding "directionality" of the voxels' signals (above or below average) rather than their absolute amplitude strength, we chose to use the cosine similarity, which is invariant to the scaling of the data. In other words, excluding anticorrelated patterns, we tried to prevent spatially similar patterns in different phases and with different signal amplitudes from going into different clusters. The Pearson correlation coefficient and cosine similarity are related measures, but the Pearson correlation is also invariant to adding any constant to all data elements, which we considered to possibly have a global signal regression (GSR) type of effect on clustering (Manning et al., 2008; Murtagh & Contreras, 2012; Singhal, 2001).

A cosine similarity matrix was converted to a distance matrix, as we performed hierarchical clustering using R fastcluster‐package function hclust (method = "ward.D2") (Müllner, 2013). The results from 30 to 2 clusters (in total, 58 clusters or CAPs) were evaluated. We aggregated the fMRI volumes assigned to each cluster. The mean image of such a cluster's volumes provided an overall view of the resulting CAP and was then normalized by the standard error (within‐cluster and across fMRI volumes) to generate *z*‐statistic maps, which quantify the degree of significance to which the CAP map values for each voxel deviate from zero (Liu et al., 2013, 2018).

### 2.5 Group comparison *t* tests

The 11,930 RS‐fMRI volumes concatenated into one file were used as input for FSL's randomise (Anderson & Robinson, 2001; Winkler et al., 2014). The voxel‐wise differences between the ASD and TD groups were assessed for each CAP using two‐sample unpaired *t* tests (10,000 permutations). The design matrix for each hierarchy level included all the volumes as rows and all the clusters, that is, CAPs existing at that level of the hierarchy, as columns, with separate columns for the TD and ASD participants. We created within‐group and between‐group contrast files for the CAPs and used participant‐wise exchangeability block labels. The resulting threshold‐free cluster enhancement (TFCE) uncorrected *p*‐value maps were merged, and the false discovery rate (FDR) corrected across all the contrasts using FSL's fdr (q = 0.05), which gave a *p*‐value threshold of .004, corrected for two‐tailed results at *p* <.002 (Anderson & Robinson, 2001; Benjamini & Hochberg, 1995; Genovese et al., 2002). We used MRIcron (Rorden & Brett, 2000) and R packages ape (Paradis & Schliep, 2018), dendextend (Galili, 2015), dendsort (Sakai et al., 2014), dplyr (Wickham et al., 2020), ggtree (Yu et al., 2017), ggplot2 (Wickham, 2016), gplots (Warnes et al., 2020), plyr (Wickham, 2011), and RColorBrewer (Neuwirth, 2014) to aid data visualization.

## 3 RESULTS

**FIGURE 3:**
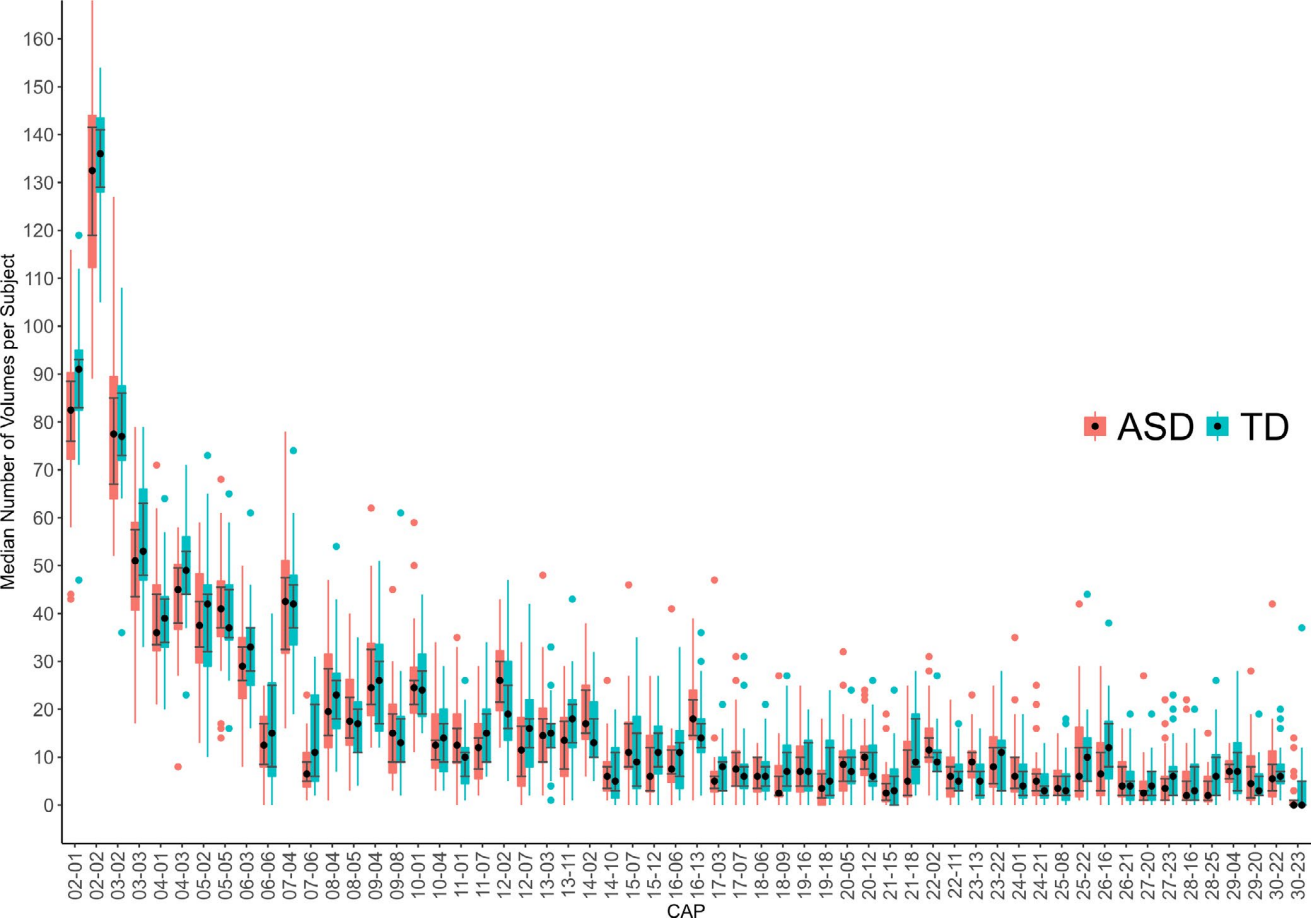
The median number of fMRI volumes per subject in CAPs with confidence intervals and outlier dots. The outliers may reflect individual differences in time spent in default mode‐positive or task‐positive brain states. As described earlier, participant‐wise, censoring excluded on average 13 volumes more from the ASD data than from the TD data

The median number of fMRI volumes assigned to each CAP from either the ASD or the TD participants' data was calculated with bootstrapped confidence intervals (95%, 10,000 resamples), which were overlapping (Figure 3). The Mann–Whitney test *p*‐value was <.05 in seven CAPs but became nonsignificant after FDR correction. Thus, we found no reliable ASD‐ or TD‐specific CAPs within the cluster levels used in our study (from 2 to 30). The number of volumes in each CAP (Figures S1a‐b) and the images of each CAP (Figures S2a‐i), and when found, their group activation differences (Figures S2a‐i), are visualized in Appendix S1.

The clustering of the whole RS‐fMRI data reveals a familiar‐looking division into two main cluster groups. At the highest level of the cladogram (Figure 1), the first level of branching, there are only two CAPs, and each fMRI volume belongs to one or the other. The first CAP (02–01, 39% of the 11,930 volumes) resembles a task‐positive ICN as its fMRI volumes contain below‐average values in lateral visual, default mode, language, and frontoparietal cognitive control (FPC) networks, but above‐average values in dorsal attention (DAN), medial visual, somatosensory, motor, auditory, salience, and ventral attention (VAN) networks. The second CAP (02–02, 61% of the volumes) shows a reversal in the activity patterns with above‐average values in default mode network (DMN) and corresponding opposite values in other networks embodying task‐negative, or rather default mode‐positive ICN features, compared to the first CAP.

**FIGURE 4:**
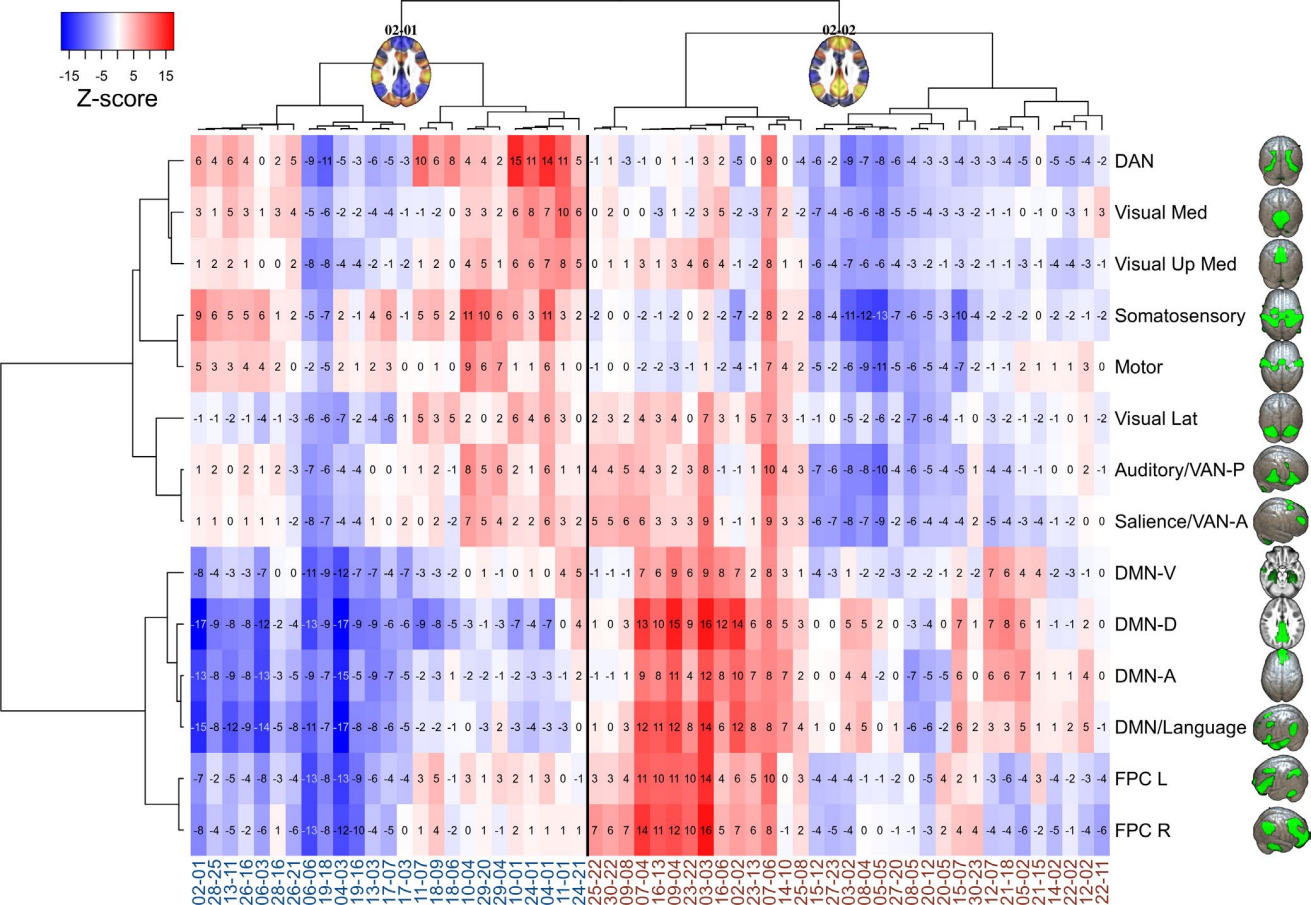
Mean voxel‐wise *z*‐scores by resting state networks (as in Figure 2) for each CAP. On the *x* and *y* axes, the brain areas' grouping was determined by the hierarchical clustering (cosine distance, Ward's method) of only this particular *z*‐score matrix. On the *x*‐axis, the CAP label colors correspond to the "DMN‐negative/task‐positive" (blue labels, DMN‐CAPs) and "DMN‐positive" (red labels, DMN + CAPs) grouping of CAPs, with a thin black separating line between them. Network abbreviations: DAN = dorsal attention; Visual Med, Up Med, Lat = visual medial, upper medial, lateral; VAN‐P/‐A = ventral attention posterior/anterior; DMN‐V/‐D/‐A = default mode ventral/dorsal/anterior, FPC L/R = frontoparietal cognitive control left/right. Views: DAN, Visual (3D elevated occipital), Somatosensory, Motor, DMN‐A (3D above), Auditory, Salience, VAN, FPC R (3D right lateral), DMN‐V, ‐D (axial slice above), DMN/Language, FPC L (3D left lateral)

Both DMN‐positive and DMN‐negative (or task‐positive) CAPs divide into subhierarchies and smaller CAPs (Figure 1), which are distinguished from each other by different areal average values (also referred to here as activation when above average and deactivation when below average) in ICA‐based RSNs (Figure 2). This is also depicted in the heatmap of Figure 4, which illustrates the average *z*‐score values of our study‐specific RSN parcellations in different CAPs. The hierarchical clustering of both rows and columns of this heatmap groups the different RSNs and CAPs by their features, respectively. This kind of review is modest in terms of spatial accuracy but facilitates pattern recognition in group‐level activation differences. The most obvious patterns are reported in the following paragraphs.

When the CAPs in Figure 1 are transformed into the heatmap of Figure 4, one can roughly detect four panels. Firstly, the DMN, language, and FPC networks exhibit mainly negative *z*‐scores (interpreted here as network deactivation) during the default mode‐negative CAPs (DMN‐CAPs) and form the bluish colored lower panel on the left, whereas the DAN and all the sensory, motor, salience, and VAN networks mostly exhibit red colored positive *z*‐scores (interpreted here as task‐positive network activation) and form the reddish left upper panel of the DMN‐CAPs.

But during the DMN‐positive CAPs (DMN + CAPs) on the right, the DMN, language, and FPC networks largely exhibit positive *z*‐scores and form the reddish right lower panel. Fourthly, the right upper panel of the DMN + CAPs consist for the most part, of blue colored negative *z*‐scores, excluding a few CAPs with activation among auditory, visual, salience, and VAN networks.

**FIGURE 5:**
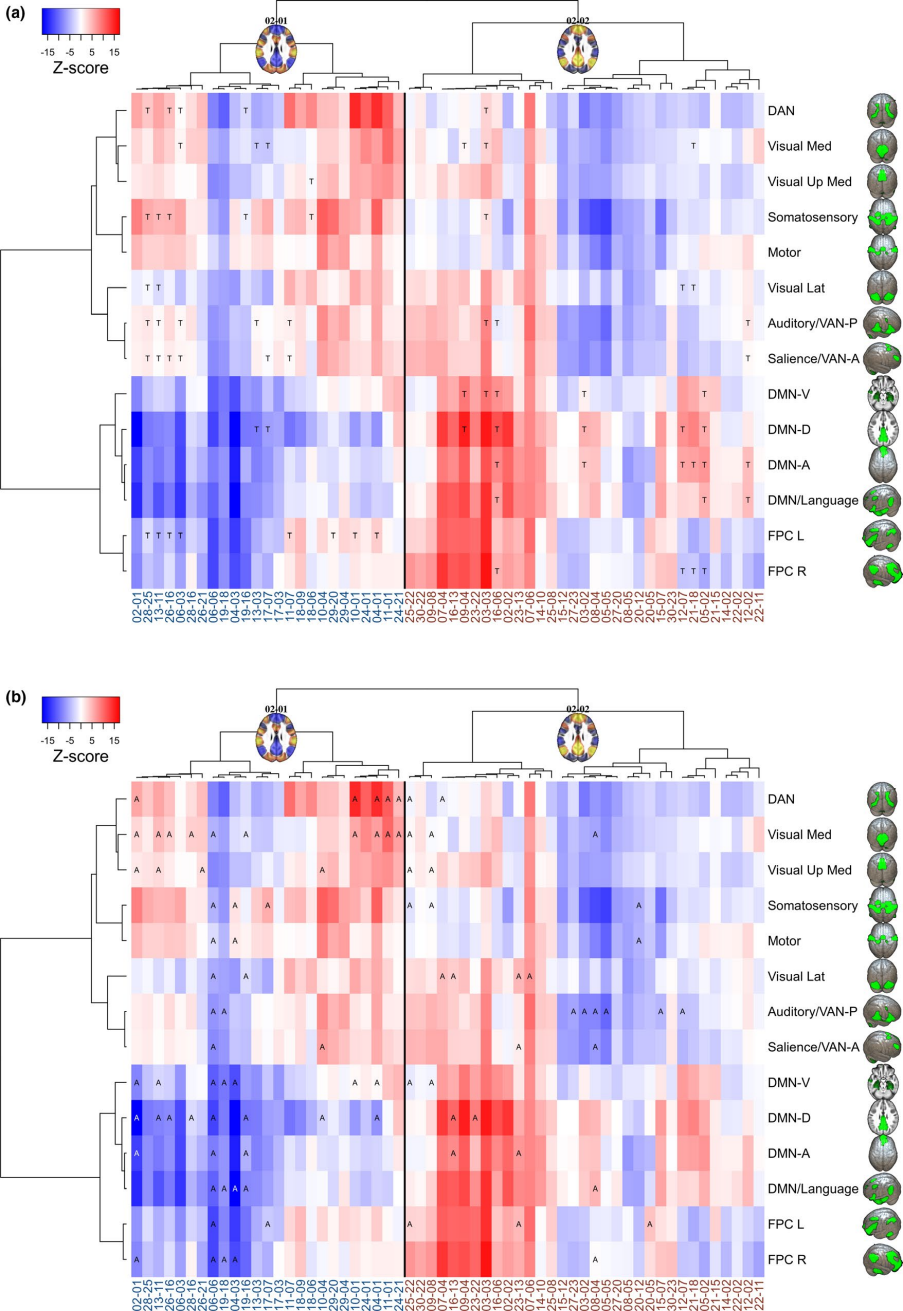
Combination of Figures 4 and 6. Largest clusters (highest decile) of TD and ASD group‐related CAP activity in resting state networks. (a) The TD group‐related activity is marked by the letter "T" and (b) the ASD group‐related activity by "A" over the color‐coded average *z*‐scores of the same heatmap as those in Figure 4

Due to *z*‐score averaging, the nuances of the CAPs are lost in the heatmap, and a few seemingly similar CAPs coexist on both left and right. Notably, some CAPs also show only activation or deactivation over all the RSNs, that is, negative blue or positive red vertical stripes over the whole brain cortex matrix (Figures 4 and 5).

**FIGURE 6:**
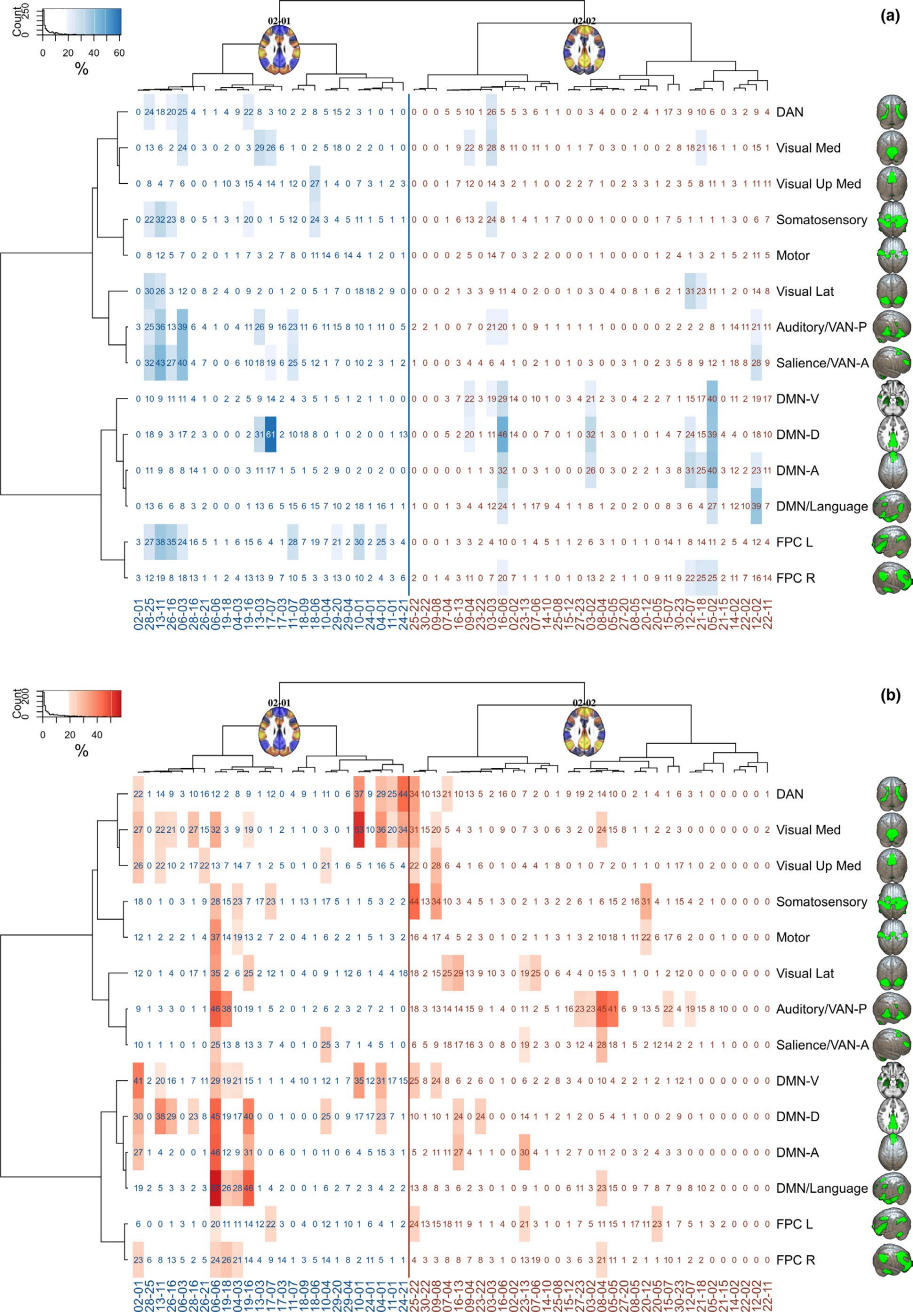
Percentages of brain areas in CAPs in which the (a) TD and (b) ASD groups have significantly greater voxel‐wise *z*‐score values than the other group. The results are shown at q = .05 false discovery rate (FDR) corrected. An arbitrary threshold of the highest decile is applied to the cell background coloring (blue rectangles for TD, red for ASD) as a highlighting method, in which cluster areas comprise at least 19% of the corresponding IC areas. The order and origin of the *x*‐ and *y*‐axis labels are identical to those in Figure 4

The areas in the CAPs in which the TD or ASD group have significantly greater activation than the other group are shown as a percentage of the corresponding RSN volume in Figure 6. The highlighted highest deciles of these results are projected on the earlier CAP heatmap (Figure 4). This combination in Figure 5 enables finding the RSNs in the CAPs that exhibit the largest simultaneous group‐related activations. The twenty largest between‐group differences in CAPs are listed in Table 1. Additional descriptive (Figures S1a‐b, Figures S2a‐i) and detailed results Table S1 can be found in Appendix S1 of this article and in Zenodo (Paakki et al., 2021), respectively.

**TABLE 1:** Twenty biggest CAP group differences in FSL randomise TFCE clusters (false discovery rate q < 0.05 corrected, two‐tailed FDR‐adjusted *p*‐value threshold *p* =.002) and percentage of brain areas in each CAP and IC in which the ASD or TD groups had significantly greater values than the other group

CAP		VOXELS	CLUSTERS	ICA‐AREAS by % (voxels of clusters area/ICA area)
06–06	ASD > TD	9,565	1	DMN/Language, 57% (1298/2259); DMN‐A, 46% (900/1951); Auditory/VAN‐P, 46% (1086/2356); DMN‐D, 45% (419/926); Motor, 37% (1113/2998); Visual Lat, 35% (530/1518); Visual Med, 32% (766/2375); DMN‐V, 29% (454/1581); Somatosensory, 28% (759/2716); Salience/VAN‐A, 25% (643/2523); FPC R, 24% (806/3403); FPC L, 20% (423/2143); Visual Up Med, 13% (149/1150); DAN, 12% (212/1769);
13–11	TD > ASD	6,027	9	Salience/VAN‐A, 43% (1087/2523); FPC L, 38% (819/2143); Auditory/VAN‐P, 36% (846/2356); Somatosensory, 32% (873/2716); Visual Lat, 26% (390/1518); FPC R, 19% (636/3403); DAN, 18% (310/1769); Motor, 12% (362/2998); DMN‐V, 9% (136/1581); DMN‐D, 9% (80/926); DMN‐A, 9% (168/1951); Visual Med, 6% (133/2375); DMN/Language, 6% (143/2259); Visual Up Med, 4% (44/1150);
02–01	ASD > TD	5,631	14	DMN‐V, 41% (653/1581); DMN‐D, 30% (278/926); Visual Med, 27% (650/2375); DMN‐A, 27% (529/1951); Visual Up Med, 26% (298/1150); FPC R, 23% (797/3403); DAN, 22% (394/1769); DMN/Language, 19% (420/2259); Somatosensory, 18% (484/2716); Visual Lat, 12% (175/1518); Motor, 12% (355/2998); Salience/VAN‐A, 10% (244/2523); Auditory/VAN‐P, 9% (223/2356); FPC L, 6% (119/2143);
25–22	ASD > TD	5,618	15	Somatosensory, 44% (1185/2716); DAN, 34% (608/1769); Visual Med, 31% (739/2375); DMN‐V, 25% (392/1581); FPC L, 24% (504/2143); Visual Up Med, 22% (251/1150); Visual Lat, 18% (268/1518); Auditory/VAN‐P, 18% (423/2356); Motor, 16% (468/2998); DMN/Language, 13% (289/2259); DMN‐D, 10% (96/926); Salience/VAN‐A, 6% (158/2523); DMN‐A, 5% (106/1951); FPC R, 4% (131/3403);
06–03	TD > ASD	5,476	9	Salience/VAN‐A, 40% (1014/2523); Auditory/VAN‐P, 39% (930/2356); DAN, 25% (451/1769); Visual Med, 24% (568/2375); FPC L, 24% (513/2143); FPC R, 18% (623/3403); DMN‐D, 17% (158/926); Visual Lat, 12% (180/1518); DMN‐V, 11% (171/1581); Somatosensory, 8% (220/2716); DMN/Language, 8% (190/2259); DMN‐A, 8% (162/1951); Motor, 7% (222/2998); Visual Up Med, 6% (72/1150);
19–16	ASD > TD	5,445	8	DMN/Language, 46% (1040/2259); DMN‐D, 40% (373/926); DMN‐A, 31% (604/1951); Visual Lat, 25% (382/1518); Visual Med, 19% (462/2375); Auditory/VAN‐P, 19% (440/2356); DMN‐V, 15% (235/1581); FPC R, 14% (463/3403); FPC L, 14% (294/2143); Salience/VAN‐A, 13% (335/2523); Motor, 13% (375/2998); DAN, 9% (157/1769); Visual Up Med, 7% (82/1150); Somatosensory, 7% (191/2716);
28–25	TD > ASD	5,340	8	Salience/VAN‐A, 32% (819/2523); Visual Lat, 30% (460/1518); FPC L, 27% (569/2143); Auditory/VAN‐P, 25% (588/2356); DAN, 24% (418/1769); Somatosensory, 22% (590/2716); DMN‐D, 18% (163/926); Visual Med, 13% (313/2375); DMN/Language, 13% (295/2259); FPC R, 12% (414/3403); DMN‐A, 11% (207/1951); DMN‐V, 10% (166/1581); Visual Up Med, 8% (94/1150); Motor, 8% (244/2998);
08–04	ASD > TD	5,248	12	Auditory/VAN‐P, 45% (1057/2356); Salience/VAN‐A, 28% (713/2523); Visual Med, 24% (574/2375); DMN/Language, 23% (519/2259); FPC R, 21% (729/3403); Visual Lat, 15% (231/1518); DAN, 14% (254/1769); FPC L, 11% (226/2143); DMN‐A, 11% (208/1951); Motor, 10% (305/2998); DMN‐V, 10% (158/1581); Visual Up Med, 7% (77/1150); Somatosensory, 6% (151/2716); DMN‐D, 5% (46/926);
12–02	TD > ASD	5,126	8	DMN/Language, 39% (875/2259); Salience/VAN‐A, 28% (699/2523); DMN‐A, 23% (455/1951); Auditory/VAN‐P, 21% (484/2356); DMN‐V, 19% (294/1581); DMN‐D, 18% (166/926); FPC R, 16% (545/3403); Visual Med, 15% (363/2375); Visual Lat, 14% (217/1518); FPC L, 12% (257/2143); Visual Up Med, 11% (124/1150); Motor, 11% (332/2998); DAN, 9% (155/1769); Somatosensory, 6% (158/2716);
05–02	TD > ASD	4,863	9	DMN‐V, 40% (632/1581); DMN‐A, 40% (776/1951); DMN‐D, 39% (364/926); DMN/Language, 27% (603/2259); FPC R, 25% (859/3403); Visual Med, 16% (384/2375); Salience/VAN‐A, 12% (301/2523); Visual Up Med, 11% (123/1150); Visual Lat, 11% (168/1518); FPC L, 11% (246/2143); Auditory/VAN‐P, 8% (180/2356); DAN, 6% (114/1769); Motor, 2% (67/2998); Somatosensory, 1% (36/2716);
19–18	ASD > TD	4,693	19	Auditory/VAN‐P, 38% (907/2356); FPC R, 26% (894/3403); DMN/Language, 26% (579/2259); DMN‐V, 19% (299/1581); DMN‐D, 19% (173/926); Somatosensory, 15% (412/2716); Motor, 14% (425/2998); Salience/VAN‐A, 13% (317/2523); DMN‐A, 12% (228/1951); FPC L, 11% (234/2143); Visual Up Med, 7% (78/1150); Visual Med, 3% (76/2375); Visual Lat, 2% (36/1518); DAN, 2% (35/1769);
09–08	ASD > TD	4,525	13	Somatosensory, 34% (915/2716); Visual Up Med, 28% (321/1150); DMN‐V, 24% (375/1581); Visual Med, 20% (481/2375); Motor, 17% (502/2998); Visual Lat, 15% (231/1518); FPC L, 15% (322/2143); DAN, 13% (223/1769); Auditory/VAN‐P, 13% (304/2356); DMN‐A, 11% (220/1951); DMN‐D, 10% (93/926); Salience/VAN‐A, 9% (236/2523); DMN/Language, 8% (188/2259); FPC R, 3% (108/3403);
04–03	ASD > TD	4,472	18	DMN/Language, 28% (630/2259); Somatosensory, 23% (614/2716); FPC R, 21% (708/3403); DMN‐V, 21% (328/1581); Motor, 19% (568/2998); DMN‐D, 17% (156/926); Visual Up Med, 14% (157/1150); FPC L, 11% (227/2143); Auditory/VAN‐P, 10% (236/2356); Visual Med, 9% (223/2375); DMN‐A, 9% (185/1951); Salience/VAN‐A, 8% (200/2523); DAN, 8% (143/1769); Visual Lat, 6% (89/1518);
16–06	TD > ASD	4,426	35	DMN‐D, 46% (426/926); DMN‐A, 32% (622/1951); DMN‐V, 29% (459/1581); DMN/Language, 24% (542/2259); FPC R, 20% (695/3403); Auditory/VAN‐P, 20% (477/2356); Visual Lat, 11% (163/1518); FPC L, 10% (210/2143); Visual Med, 8% (179/2375); Somatosensory, 8% (216/2716); Motor, 7% (213/2998); DAN, 5% (81/1769); Salience/VAN‐A, 4% (103/2523); Visual Up Med, 3% (39/1150);
03–03	TD > ASD	4,187	17	Visual Med, 28% (667/2375); DAN, 26% (452/1769); Somatosensory, 24% (652/2716); Auditory/VAN‐P, 21% (487/2356); DMN‐V, 19% (301/1581); Visual Up Med, 14% (160/1150); Motor, 14% (426/2998); DMN/Language, 12% (263/2259); DMN‐D, 11% (103/926); Visual Lat, 9% (134/1518); FPC R, 7% (246/3403); Salience/VAN‐A, 6% (140/2523); FPC L, 4% (94/2143); DMN‐A, 3% (62/1951);
05–05	ASD > TD	4,151	20	Auditory/VAN‐P, 41% (971/2356); Salience/VAN‐A, 18% (450/2523); Motor, 18% (534/2998); Visual Med, 15% (358/2375); Somatosensory, 15% (401/2716); FPC L, 15% (319/2143); DMN/Language, 15% (345/2259); FPC R, 11% (384/3403); DAN, 10% (173/1769); DMN‐V, 4% (69/1581); DMN‐D, 4% (33/926); Visual Lat, 3% (51/1518); Visual Up Med, 2% (26/1150); DMN‐A, 2% (37/1951);
26–16	TD > ASD	3,768	16	FPC L, 35% (748/2143); Salience/VAN‐A, 27% (682/2523); Somatosensory, 23% (620/2716); DAN, 20% (351/1769); Auditory/VAN‐P, 13% (296/2356); DMN‐V, 11% (176/1581); FPC R, 8% (257/3403); DMN‐A, 8% (149/1951); Visual Up Med, 7% (83/1150); DMN/Language, 6% (131/2259); Motor, 5% (162/2998); Visual Lat, 3% (42/1518); DMN‐D, 3% (25/926); Visual Med, 2% (46/2375);
21–18	TD > ASD	3,663	18	FPC R, 25% (866/3403); DMN‐A, 25% (483/1951); Visual Lat, 23% (345/1518); Visual Med, 21% (498/2375); DMN‐V, 17% (273/1581); DMN‐D, 15% (141/926); FPC L, 14% (291/2143); DAN, 10% (179/1769); Salience/VAN‐A, 9% (238/2523); Visual Up Med, 8% (89/1150); DMN/Language, 4% (86/2259); Motor, 3% (80/2998); Auditory/VAN‐P, 2% (42/2356); Somatosensory, 1% (39/2716);
13–03	TD > ASD	3,564	6	DMN‐D, 31% (289/926); Visual Med, 29% (681/2375); Auditory/VAN‐P, 26% (609/2356); Salience/VAN‐A, 18% (455/2523); FPC R, 13% (434/3403); DMN/Language, 13% (300/2259); DMN‐A, 11% (217/1951); DMN‐V, 9% (144/1581); DAN, 8% (134/1769); FPC L, 6% (131/2143); Visual Up Med, 4% (43/1150); Motor, 3% (83/2998); Visual Lat, 2% (31/1518); Somatosensory, 0% (11/2716);
12–07	TD > ASD	3,538	21	Visual Lat, 31% (466/1518); DMN‐A, 31% (608/1951); DMN‐D, 24% (220/926); FPC R, 22% (733/3403); Visual Med, 18% (421/2375); DMN‐V, 15% (233/1581); DAN, 9% (165/1769); Salience/VAN‐A, 8% (213/2523); FPC L, 8% (170/2143); DMN/Language, 6% (134/2259); Visual Up Med, 5% (63/1150); Auditory/VAN‐P, 3% (59/2356); Somatosensory, 1% (21/2716); Motor, 1% (23/2998);

When inspecting Figure 5 through the four‐panel approach described earlier, one can see that most of the largest TD group‐related activations fall within the task‐related networks in the upper left panel during the DMN‐CAPs. In contrast, during the DMN + CAPs, the TD group‐related activations are mostly within the DMN and the language and VAN networks.

The TD group‐related FPC activation was left‐dominant during the DMN‐CAPs and right‐dominant during the DMN + CAPs. The left FPC was activated, while the task‐related networks were also activated and seemed to associate with the salience and VAN‐A networks in particular, but also with the auditory, somatosensory, and DAN networks. Right FPC activation associated with the DMN and language networks.

The earlier described four‐panel approach generally showed more incoherently highlighted patterns during the ASD group‐related activations than the TD group‐related activations (Figures 5 and 6). The ASD group's FPC activations did not demonstrate clear‐cut sidedness related to the DMN‐negative or DMN‐positive CAPs. However, there were some repeating overactivation patterns. The DAN associated with the visual networks. If the visual network was overactive but not concurrent with the DAN, it associated in turn with the DMN. In the TD group, the DAN overactivation was also associated with other task‐positive networks in a more balanced way. It also seems that the ASD group did not quite reach as strong deactivations as the TD group (Figure 5). ASD group‐related overactivations were found especially in the DAN, visual and auditory networks, and the DMN. In the 06–06 CAP, which deactivated all 14 RSNs, the ASD group showed a considerably reduced deactivation pattern in nearly all (12/14) of the RSNs.

## 4 DISCUSSION

### 4.1 Study results and comparison to earlier studies

Our study provides complementary information and an alternative perspective to FC analysis by gathering nonsequentially brief instances of similar fMRI brain volumes into larger CAP clusters. This method may be especially beneficial before group comparisons in RS studies, in which no external synchronization is provided by tasks or stimuli. We found the DMN + CAPs and DMN‐CAPs (61% and 39% of the fMRI volumes, respectively) to be spatially similar in both TD and ASD groups as the clustering algorithm gathered volumes to each CAP from both groups without significant group‐wise differences in time spent in each CAP. However, the CAPs showed focused alterations of internal activity levels among many RSNs, including the following (Figures 5 and 6):

1) ASD‐related activations during the DMN‐CAPs considerably affected the DMN, and during DMN + CAPs, other RSNs.

2) The ASD group showed visual network overactivation during the DMN‐CAPs, which was simultaneous with the overactivation of either the DAN or the DMN.

3) Autism spectrum disorder‐related FPC activations were incoherent and showed hemispherical shifts.

4) The auditory, DMN, and language networks were overactivated in the ASD group during the RSN deactivations, which may indicate higher baseline activity in the ASD group.

The TD group‐related alterations can be assessed similarly: In general, the TD group showed greater activation in the task‐positive RSNs during the DMN‐CAPs and in the positively activated DMN during the DMN + CAPs, than the ASD group. During the DMN‐CAPs, auditory activation reached higher levels in the TD group. In addition, DAN activation was also more evenly related to other sensory (auditory, somatosensory), salience, and VAN networks. FPC overactivation was consistently asymmetric in the TD group: predominantly left‐sided during the DMN‐CAPs and right‐sided during the DMN + CAPs.

Comparing the group‐related changes in Figure 5 shows that the ASD participants demonstrated overactivation of visual medial areas during the DMN‐CAPs. Simultaneous overactivation with a visual network was detected among the DAN and/or DMN. This tendency might have been related to increased reliance on posterior brain areas in ASD when mediating visuospatial tasks (Kana et al., 2013). In a recent magnetoencephalography study, the ASD group presented early enhanced activity in the occipital region, suggesting that impaired face processing in ASD might be sustained by atypical responses in primary visual areas (Kovarski et al., 2019). Anecdotal experiences of individuals with ASD report overwhelming sensations of visual details in everyday environments that they cannot pass without becoming absorbed in them. Abnormal simultaneous overactivation of the visual networks with the DAN and the DMNs detected in our study could reflect such propensity.

People with ASD experience trouble filtering torrents of information, which hijacks their concentration, and this may explain why prolonged simultaneous multisensory events cause fatigue sooner for people with ASD. On the other hand, it could be speculated that neurotypical people may not be able to concentrate with similar intensity or for equal periods under normal levels of stimuli. It would be interesting to study further whether the neurobiological potential for concentration in ASD could be reflected in task‐related DMN activation. Such a study could utilize the gradual‐onset continuous performance task (gradCPT). The gradCPT is a sustained attention ability paradigm that has been validated behaviorally and recently with neuroimaging (Fortenbaugh et al., 2018). The gradCPT results show that fluctuations in attentional stability are tracked over time in task‐positive (e.g., DAN and VAN) and task‐negative (e.g., DMN) regions and vary in specific ways before attention lapses or concerning reaction time and performance. The DMN contributions are not unambiguous but also modulated, for example, by motivation (Fortenbaugh et al., 2018). We are not aware of ASD‐related studies utilizing gradCPT and fMRI.

In healthy adults, a network graph study found that optimal sustained attention arose from reduced network cross talk and greater within‐network communication in task‐relevant networks such as salience, cingulo‐opercular, dorsal attention, and visual (Zuberer et al., 2021). In contrast, optimal attention predicted greater network cross talk and reduced within‐network communication in auditory and sensorimotor networks and lower within‐network communication in the subcortical and ventral attention networks (Zuberer et al., 2021). The relationship between network graphs and brain (de)activations needs clarification.

Concentration or sustained attention and mind‐wandering or task‐unrelated thoughts are two general mind states alternating with varying frequency and duration during some task. In a study by Scheibner et al. (2017), mindful attention was characterized by less activity in the DMN than mind‐wandering, independent of attention type (internal breathing or external sound). The activation difference was greater in the inner attention meditation than in the external attention meditation. While the ability to concentrate or uphold sustained attention is not equal to mindfulness, these concepts are related, and mindfulness‐based interventions can increase attention (Trautwein et al., 2020). These transient cognitive states may be captured better in dynamical temporal analysis than static methods. Marusak et al. (2018) showed that trait mindfulness in youths related to dynamic but not static RS connectivity. The more mindful youths transitioned more between brain states, spent less time in a particular connectivity state, and showed a state‐specific reduction in connectivity between salience and central executive (i.e., frontoparietal cognitive control) networks (Marusak et al., 2018). In our study, the strongest salience and FPC mean activations were detected during DMN + CAPs (Figure 4). We found TD group‐related salience and FPC L association during the DMN‐CAPs, but during the DMN + CAPs, similar activity was seen in two ASD‐related CAPs only (Figures 5 and 6). The inferences between FC and CAP analysis are not yet clear.

As stated earlier, in the ASD group, during the DAN activated task‐positive CAPs (24–21, 13–11, 11–01, 10–01, 04–01, and 02–01, Appendix S1: Figures S2a‐i), there was a repeated association with ASD‐related overactivation of the visual medial network (Figure 5). Parallel top‐down volitional attention is influenced by the DAN, which has key nodes in the bilateral intraparietal sulcus, superior parietal lobule, and frontal eye fields (Vossel et al., 2014; Yamasaki et al., 2017). Research has demonstrated that these dorsal frontoparietal areas can causally modulate visual areas' activity (Vossel et al., 2014). One hypothetical explanation for our study results could be that this modulating effect may be more substantial among ASD individuals. However, we detected visual overactivation during many DMN‐CAPs, and the CAP method used here cannot infer causality. Yamasaki et al. (2017) reviewed studies using visual evoked potentials, event‐related potentials, and the diffusion tensor MRI of visual and attention networks in ASD. They found that (1) enhanced and impaired processing coexists within the lower visual area (V1), (2) local information integration from lower visual areas (V1) is impaired in higher‐level visual areas after V4 and V5/MT, and (3) the DAN is impaired, while the VAN is intact in ASD. The VAN contains key nodes in the temporoparietal junction and ventral frontal cortices related to automatically produced and quicker bottom‐up attention (Yamasaki et al., 2017). Despite the results of Yamasaki et al. (2017), some fMRI studies have found ASD‐related abnormalities in the VAN as well (Bernas et al., 2018; Farrant & Uddin, 2016; Fitzgerald et al., 2015).

Moreover, a study by Feczko et al. (2018) hints that some ASD subgroups have altered visual processing or attention mechanisms or both. In addition to overwhelming sensory experiences, altered connectivity of visual and attention networks may contribute to the impaired social communication in ASD. Early disordered FC involving the visual network may engender later disruptions in higher order behaviors. McKinnon et al. (2019) showed that aberrant functional connectivities between the visual, control, DMN, DAN, and subcortical networks are also associated with certain restricted and repetitive behaviors among children with ASD at 12 and 24 months of age. Other recent findings regarding abnormal attention mechanisms in ASD have been made in studies by Bi et al. (2018), Fitzgerald et al. (2015), and Gabrielsen et al. (2018). While ASD may offer advantages in various visual‐attentional tasks, the predisposition to intense attentional focus may come at the cost of resistance to task disengagement and other behavioral symptoms such as overfocusing and restricted interests (Kaldy et al., 2016).

In the TD group, highlighted simultaneous DAN and visual network overactivation were detected only during task‐positive CAP 06–03 and DMN‐positive CAP 03–03. The former, unlike the CAPs mentioned in the previous paragraph, exhibits mainly motor and somatosensory activation. In addition to the DAN and visual medial networks, TD group‐related overactivation is more comprehensive and detected among the auditory, salience and VAN, and FPC networks.

A recent study showed that tactile and auditory hypersensitivity among children raised the risk of ASD diagnosis 34‐ and 22‐fold, respectively (Jussila et al., 2019). Our results suggest higher auditory network baseline activity during deactivations in ASD and that somatosensory activations are less unambiguous. Despite hearing protection, noisy MRI environments may cause more auditory than somatosensory input in a supine patient lying still.

Autism spectrum disorder‐related functional brain asymmetry has been detected during RS by, for example, Cardinale et al. (2013) and Subbaraju et al. (2018), who have shown rightward asymmetry shifts of functional networks and atypical hemispherical lateralization, respectively. Diffusion imaging has found inversion or diminishing of typical left‐right asymmetry among ASD individuals (Carper et al., 2016; Conti et al., 2016; Wei et al., 2018). In a similar vein, our study demonstrated ASD group‐related FPC activation shifts that were rightward during the DMN‐CAPs and leftward during the DMN + CAPs (Figure 5).

Earlier evidence of reduced functional integration of the DMN, especially weaker coherence of connectivity between the posterior and anterior subsystems (Joshi et al., 2017; Starck et al., 2013), may be mirrored in our study as higher baseline activity during the DMN‐CAPs, especially in the dorsal and ventral components of DMN. Still, the inferences between FC and CAP analysis remain unclear.

### 4.2 Limitations and future directions

Resting state studies have found it difficult to show unambiguous brain FC changes in ASD (Hull et al., 2017). Even though local (e.g., regional homogeneity) and more distant changes of FC have been shown (Hull et al., 2017; Jao Keehn et al., 2018; Nair et al., 2018), legitimate concern has arisen that motion during RS examinations might at least partly explain the detected hypo‐ and/or hyperconnectivity (Jo et al., 2013; Power et al., 2014). Recent evidence suggests that censoring and ICA‐AROMA perform well across most preprocessing quality benchmarks (Parkes et al., 2018). Whereas earlier dual‐regression ICA and FC analysis has revealed only hypoconnectivity within the DMN subnetworks of our study participants with ASD (Starck et al., 2013), we found significant differences in many CAPs. When similarly activated brain BOLD fMRI volumes are accumulated into CAPs, between‐group comparisons may become more powerful than, for example, sliding window methods, in which each "window" of sequential volumes includes more heterogeneous brain activation patterns. When discussing results, one should remember that hierarchical clusters are nested and thus volumes accumulate as we move up the hierarchy into lower‐numbered cluster levels. Depending on the different spatial (de)activation signal amplitudes of the clusters (CAPs) combined and the difference in the brain areas' activation behavior between the groups, some of the spatial between‐group differences may fade, and others may increase from one hierarchical level to another.

It should be remembered that hierarchical clustering is an exploratory method and imposes a hierarchical structure regardless of whether such exists in the data (Friedman et al., 2001). Accordingly, the results should be interpreted cautiously. However, based on both previous research knowledge about alternating rest and task states of brain function and our results, this method can yield meaningful complementary information on the "natural" occurrence of CAPs and their relations to each other during RS‐fMRI. The coarse division in our data showed that 61% of the volumes had default mode‐positive RSN features. RS data certainly also include varying epochs with true task‐positive ICN activations, as the MRI environment is noisy and disruptive, especially to young individuals, and we imaged RS with the participants' eyes open. Empirical evidence suggests that eyes‐open brain states are better controlled than eyes‐closed states, but that eye status affects local connectivity, highlighting overconnectivity in posterior, visual regions and underconnectivity in the cingulate gyrus (Nair et al., 2018). We did not compare RS with task data, and the relationship between the CAPs of the rest and task data should be addressed in the future to determine the proportions of DMN‐positive and task‐positive activity in combined data and the various settings: or to determine whether certain CAPs and their between‐group differences persist during tasks and how they are modified. In this context, there is suggestive evidence that functional hierarchies in the pediatric brain are stable and similar during rest and task (Harrewijn et al., 2020).

As stated earlier, due to *z*‐score averaging, the nuances of CAPs are lost in the heatmap, and a few seemingly similar CAPs coexist on both DMN‐negative and DMN‐positive sides of the clustering results. The clustering procedure itself is, of course, unaware of this interpretive naming convention and simplification aimed to facilitate the understanding of complex network interactions. In addition to averaging, the weaknesses of the chosen method may predispose to this phenomenon.

There are several linkage methods in hierarchical clustering. We chose Ward's method as it shares a common principle with k‐means, providing a basis for current research and comparison to earlier research. We found a high correlation with the CAPs from Liu et al. (2013) using the fslcc tool (results not shown), though their results were acquired after global mean removal. Using GSR could eliminate artifacts even more efficiently than censoring and ICA‐AROMA alone (Byrge & Kennedy, 2018; Ciric et al., 2017; Murphy & Fox, 2017; Power et al., 2015). Possible anticorrelations in the CAPs might not be as problematic as with FC measures, as signal amplitudes are compared. Our educated guess is that using GSR would reduce the portion of CAPs that exhibit whole brain‐wide activation or deactivation. Unfortunately, censoring reduces degrees of freedom and may also remove the signal of interest from the data. For example, Syed et al. (2017) found that although the DMN provided the highest discriminability between the control and ASD groups, the motor network regions with midcingulate cortex and temporal‐parietal junction were also discriminatory. Moreover, the choice of clustering distance measure (cosine, Euclidean, Pearson correlation, etc.) may potentially increase or decrease GSR‐like effects.

Besides distance and linkage adjustments, combining other statistical procedures such as permutational methods to hierarchical clustering could achieve results closer to the ground truth. Based on our study, however, efforts to refine volume‐wise methods are worth pursuing. Hierarchical and k‐means are only two common, older clustering methods, and more efficient algorithms that can utilize fMRI‐specific data features probably exist. For example, random forest methods could be used in a volume‐wise fashion instead of measures from temporally stationary FC (Feczko et al., 2018; Fernández‐Delgado et al., 2014). Though FC and ICA RS metrics are not substantially affected by different TRs, faster imaging methods such as MREG with 10–20 Hz temporal resolution show "neural avalanches," which in traditional 0.5–1 Hz fMRI temporal resolutions are only seen as aliased images and could enable the study of higher cluster numbers and shorter CAPs (Huotari et al., 2019; Rajna et al., 2015), though the inherently slow hemodynamic response function may act as a limiting bottleneck (Bolton et al., 2020). Faster imaging and dynamic lag analysis (Kotila et al., 2020; Raatikainen et al., 2020) or causality analysis methods (Bernas et al., 2018; Bielczyk et al., 2019; Borchers et al., 2012; Chen et al., 2016; Deshpande & Hu, 2012; Kaminski et al., 2016; Li et al., 2020) may shed light on interactions between attention, visual, and other brain networks. MREG fMRI coupled with simultaneous EEG analysis (Hiltunen et al., 2014; Li et al., 2019; Ridley et al., 2017) could clarify the relationship between the neural avalanches and the brain's electrical activity in the future.

As each volume is a time point in the imaging time series and is assigned with cluster membership, this method could map the changes at the individual level. The current study could be extended using a network or Markov chain analysis to determine whether there are repetitive sequences or states in the occurrence of the CAPs (Chen et al., 2015; Liu et al., 2013; Zhuang et al., 2018), as some ASD studies indicate (Malaia et al., 2016; Zhang et al., 2016).

Even if independent RSNs seemed to activate normally, CAP analysis might reveal aberrant in‐between network interactions and their timing. FC analysis could be supplemented by CAP analysis. It may find CAPs that exhibit the greatest differences between the voxels with aberrant connectivity and may pinpoint the moments at which the differences lie and detect simultaneous patterns in other intrinsic networks and their activity levels. This knowledge may help find new approaches to ASD rehabilitation: for example, using customized stimuli targeting brain network combinations that have been found to have abnormal interactions or appropriate timing in interaction situations.

## 5 CONCLUSION

The present study describes one relatively simple method for comparing CAPs between study populations, but because a myriad of network combinations are possible and the signal amplitude in each network varies greatly, developing a method that could satisfyingly capture the whole dynamics of brain networks is still a never‐ending challenge. Based on our study experiences, we encourage the development of volume‐wise approaches as an option to further characterize the TVFC changes in brain networks.

## CONFLICT OF INTEREST

The authors declare that the research was conducted in the absence of any commercial or financial relationships that could be construed as a potential conflict of interest.

### PEER REVIEW

The peer review history for this article is available at https://publons.com/publon/10.1002/brb3.2174.

## Supporting information

Supplementary Material

## Data Availability

Restrictions apply to the availability of the imaging data due to privacy or ethical restrictions. Data can only be shared with new permissions from the study participants.
