# Supplementary material for: Co‐activation pattern alterations in autism spectrum disorder–A volume‐wise hierarchical clustering fMRI study
Source: Brain Behav. 2021 May 16;11(6):e02174. doi: 10.1002/brb3.2174 (PMC8213933; doi:10.1002/brb3.2174)
Supplement: Supplementary file 1 — Supplementary Material [file BRB3-11-e02174-s001.docx]

**SUPPORTING INFORMATION**

**APPENDIX S1**

**MORE INFORMATION ON METHODS**

**Pre-processing of RS-fMRI signals**

For anatomical data, we used FSL-VBM FNIRT to register individual T1 structural head volumes and to generate a study-specific template (Good et al., 2001; Smith et al., 2004; Andersson et al., 2007; Douaud et al., 2007). The FSL-VBM was repeated twice using a new target template from a previous analysis. After skull stripping, the FSL's FIRST segmented the structural data into the cerebrospinal fluid (CSF), white matter (WM), and gray matter (GM) (Patenaude et al., 2011).

For the functional data, we ran the AFNI's (Cox, 1996; Cox and Hyde, 1997; Gold et al., 1998) "afni_proc.py" program to produce preprocessing pipeline scripts adapted according to the program's help page examples 9b and 10 (https://afni.nimh.nih.gov/pub/dist/doc/program_help/afni_proc.py.html) and following the guidelines of (Jo et al., 2013). We removed the first three images to avoid T1 effects and computed the outlier fractions for each volume. Motion correction was applied to align the volumes with the average of time series, and the skull was stripped. Exploiting the results of our earlier FSL-VBM procedure and the study-specific average template, we applied a non-linear transformation to functional data. The final functional volume size was 42 x 52 x 43 voxels, consisting of 4 x 4 x 4 mm voxels.

After these steps, we interrupted the AFNI pipeline and ran the independent component (IC) analysis-based automatic removal of motion artifacts (ICA-AROMA) for the functional data. After inspecting the preliminary results, we prepared custom brain edge and CSF masks fitted for our study-specific adolescent brain template. We inspected the ICA-AROMA results to exclude sporadic RSN removals. Additional ICs were removed if the IC's temporal waveform was monotonously serrated or dominated by high amplitude spikes or if the spatial distribution represented other scanner noises such as random speckles, or represented mainly white matter, arteries, vena sinuses, or edge areas. The excluded ICs were spatially cross-correlated with the FSL's fslcc among the participants and group-level ICs to keep the removal process systematic. The median number of ICs left after removal was 14.5 in ASD and 13 in the TD group (Wilcoxon rank-sum test p-value ≈ 0.41). The nonaggressive option was implemented in the participant-wise removal of artifact components (Pruim et al., 2015a, 2015b).

After ICA-AROMA, the AFNI pipeline continued with despiking. The volumes with a framewise displacement of > 0.2 mm were labeled for censoring. We calculated individual volume signal levels from the unprocessed fMRI data using the standardized version of the DVARS-script by (Nichols, 2013) and labeled the volumes with a normalized signal level of over 1.29 (outlying 10% of expected SD) for censoring (Power et al., 2014, 2015; Aurich et al., 2015). In the end, on average 13.2% (min. 0%, max. 45,6%) of the time series were censored. The shortest time left was 4 minutes 5 seconds, which was still considered adequate (White et al., 2014). After censoring, 13 750 volumes were reduced to 11 930: the TD participants had an average of 223 volumes, and the ASD participants 210 volumes left (Wilcoxon rank-sum p-value ≈ 0.34).

We extracted the Region of interest (ROI) time series regressor from the once eroded CSF mask. Although global signal regression is one of the most efficient eliminators of motion-induced artifacts (Power et al., 2014), it may remove the signal of interest and introduce artificial anti-correlations between regions (Fox et al., 2009; Gotts et al., 2013; Murphy and Fox, 2017). We were interested in the spatial similarity of the volumes (discussed later in the text) and thus did not regress the global signal. We performed spatial smoothing with 8 mm (~ 2 voxels) full width at half maximum (FWHM) kernel (Chen and Calhoun, 2018) and calculated high pass temporal filtering regressors for frequencies of < 0.005 Hz. Legendre polynomials of the 4th order were used to model slow baseline fluctuations (i.e., removal of trends). They were combined with censoring, temporal filtering, motion, CSF, and local WM (ANATICOR) regressors into a regression matrix with AFNI 3dDeconvolve. The regression matrix was then projected out of the smoothed data in one step, using the AFNI 3dTproject to remove any possible residual noise left in the data after earlier application of ICA-AROMA (Jo et al., 2010, 2013).

**Group independent component analysis (GICA)**

We created uncensored but otherwise similarly preprocessed datasets suitable for FSL MELODIC multi-session temporal concatenation analysis and estimated their group-level ICs. The IC areas were later used as voxel-wise masks and a simple atlas to label brain areas when interpreting the CAP results. We adjusted the dimensionality to 14 ICs, which in visual inspection had shown concordance with RSNs in earlier results of other groups (Smith et al., 2009; Yeo et al., 2011, 2015; Castellazzi et al., 2014; Thornburgh et al., 2017). However, dimensionality influences the naming of RSNs. Primary brain networks such as visual, auditory, motor, and somatosensory networks are quite stable, but due to low ICA dimensionality, the auditory component includes areas of the insula and temporoparietal junction, being part of salience and ventral attention networks (Vossel et al., 2014; Uddin et al., 2015). The RSN nomenclature associated with higher cognitive functions varies, and components contain overlapping areas. For example, the dorsal attention network has been addressed as the task-positive network. Moreover, the ventral attention network (VAN) can be called the executive control or central executive network or be seen as a part of the default mode or frontoparietal networks when divided into multiple components. We chose the low dimensional approach for the sake of pragmatic visual pattern analysis but still covered major networks in line with earlier studies (Starck et al., 2013; Castellazzi et al., 2014; Yeo et al., 2015; Thornburgh et al., 2017).

**Hierarchical clustering and extraction of CAPs**

The preprocessing continued in MATLAB^®^ (MathWorks^®^, 2016) and the R environments (R Core Team, 2017), as we loaded the fMRI data (Shen, 2014) into MATLAB^®^. For each participant, the fMRI signal was temporally normalized voxel-wise by subtracting the mean and then dividing by the temporal SD (Liu et al., 2018). The individual datasets were masked using combined GICA components and GM voxels (Fig. 1), which reduced the voxel count from a full cubical volume of 93 912 (42 x 52 x 43) voxels to a considerably lower 29 684 voxels and accelerated analysis. These volumes and mask were later used with FSL randomise.

The volumes were reshaped from a 3D to a 1D vector and concatenated into a 29 684 * 11 930 (voxels * volumes) matrix. The resulting data matrix from MATLAB^®^ was transferred to the R environment utilizing the R.matlab (Bengtsson, 2016) package. We applied clustering to all the BOLD fMRI volumes acquired from the 55 participants that had survived censoring. As mentioned in the introduction, the volumes are described by their voxels' signal amplitudes, and their relation to other volumes has to be defined via a suitable function. Here, individual volumes were represented as 29 684-dimensional vectors, and a matrix containing the pairwise cosine similarity among all the 11 930 vectors was calculated.

As we were interested in the spatial similarity of the volumes and the corresponding "directionality" of the voxels' signals (above or below average) rather than their absolute amplitude strength, we chose to use the cosine similarity, which is invariant to the scaling of the data. In other words, excluding anti-correlated patterns, we tried to prevent spatially similar patterns in different phases and with different signal amplitudes from going into different clusters. The Pearson correlation coefficient and cosine similarity are related measures, but the Pearson correlation is also invariant to adding any constant to all data elements, which we considered to possibly have a global signal regression (GSR) type of effect on clustering (Singhal, 2001; Manning et al., 2008; Murtagh and Contreras, 2011).

A cosine similarity matrix was converted to a distance matrix, as we performed hierarchical clustering using R fastcluster-package function hclust (method="ward.D2") (Müllner, 2013). At the level of 30 clusters, all the clusters were evaluated. For each level above this, agglomerative hierarchical clustering produced one new combined cluster from the volumes of the two existing ones, while other clusters remained unchanged until only two big clusters remained. Therefore, the results from 30 to 2 clusters and in total 58 clusters (~CAPs) were evaluated in 28 steps. After clustering, we aggregated the fMRI volumes assigned to the same cluster. The mean image of such a cluster's volumes provides an overall view of the resulting CAP. Back in MATLAB^®^, they were then normalized by the standard error (within-cluster and across fMRI volumes) to generate *z*-statistic maps, which quantify the degree of significance to which the CAP map values for each voxel deviate from zero (Liu et al., 2013, 2018).

**Group comparison t-tests**

The 11 930 RS-fMRI volumes concatenated into one file were used as input for FSL's nonparametric permutation inference tool randomise (Anderson and Robinson, 2001; Winkler et al., 2014). The voxel-wise differences between the ASD and TD groups were assessed for each CAP using two-sample unpaired t-tests with 10 000 permutations and the same mask as earlier. The design matrix for each hierarchy level included all the volumes as rows and all the clusters, that is, CAPs existing at that level of the hierarchy, as columns, with separate columns for the TD and ASD participants. We created within-group and between-group contrast files for the CAPs and used participant-wise exchangeability block labels. The resulting Threshold-Free Cluster Enhancement (TFCE) uncorrected p-value maps were merged, and the false discovery rate (FDR) corrected across all the contrasts using FSL's fdr command-line utility (q=0.05), which gave a p-value threshold of 0.004, corrected for two-tailed results at p < 0.002 (Benjamini and Hochberg, 1995; Anderson and Robinson, 2001; Genovese et al., 2002). The FDR adjusted p-value and TFCE t-statistic maps were thresholded correspondingly. Finally, it should be mentioned that we have used MRIcron (Rorden and Brett, 2000) and R packages ape (Paradis and Schliep, 2018), dendextend (Galili, 2015), dendsort (Sakai et al., 2014), dplyr (Wickham et al., 2020), ggtree (Yu et al., 2017), ggplot2 (Wickham, 2016), gplots (Warnes et al., 2020), plyr (Wickham, 2011, 20) and RColorBrewer (Neuwirth, 2014) to aid data visualization.


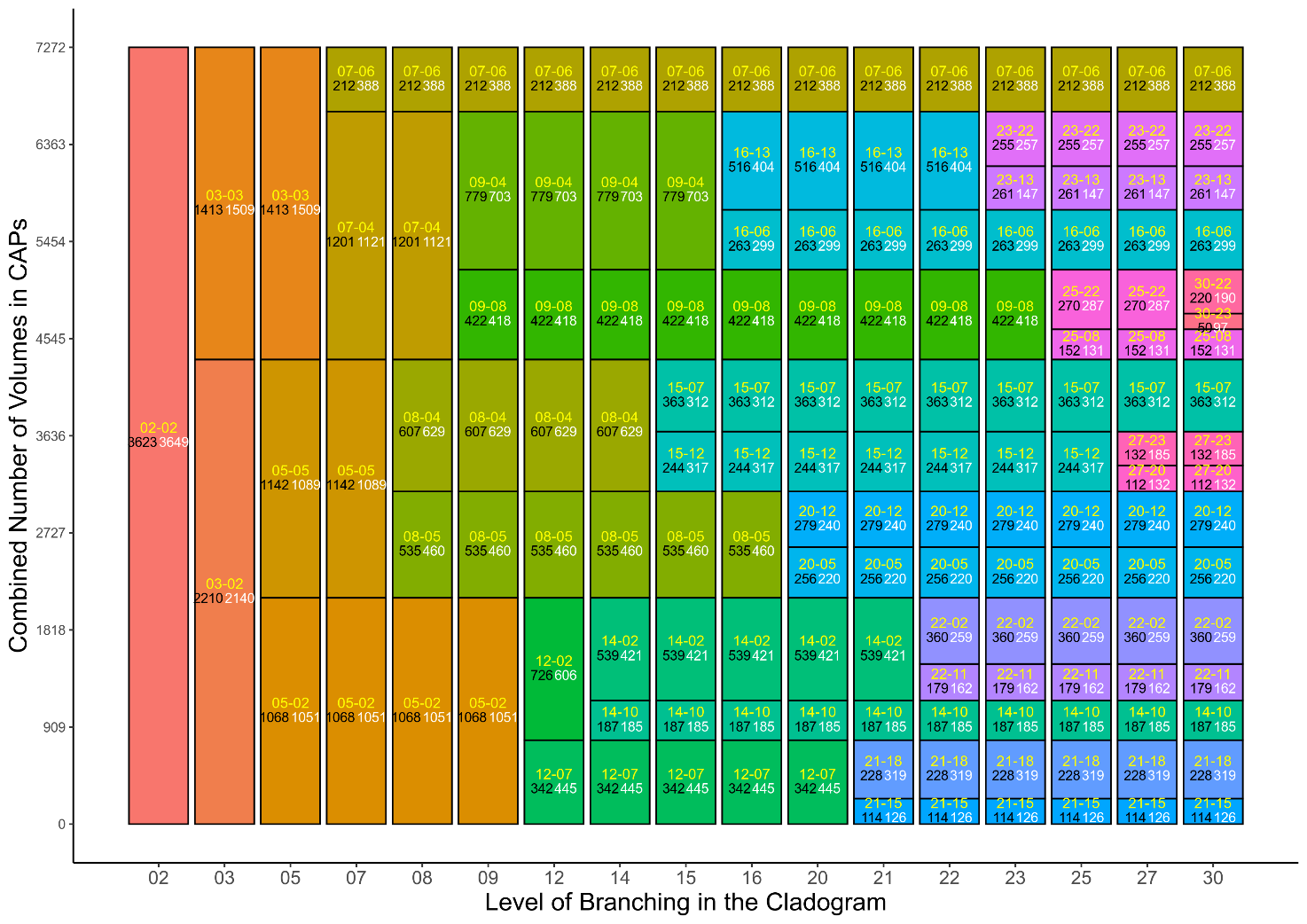


**Figure S1a.**


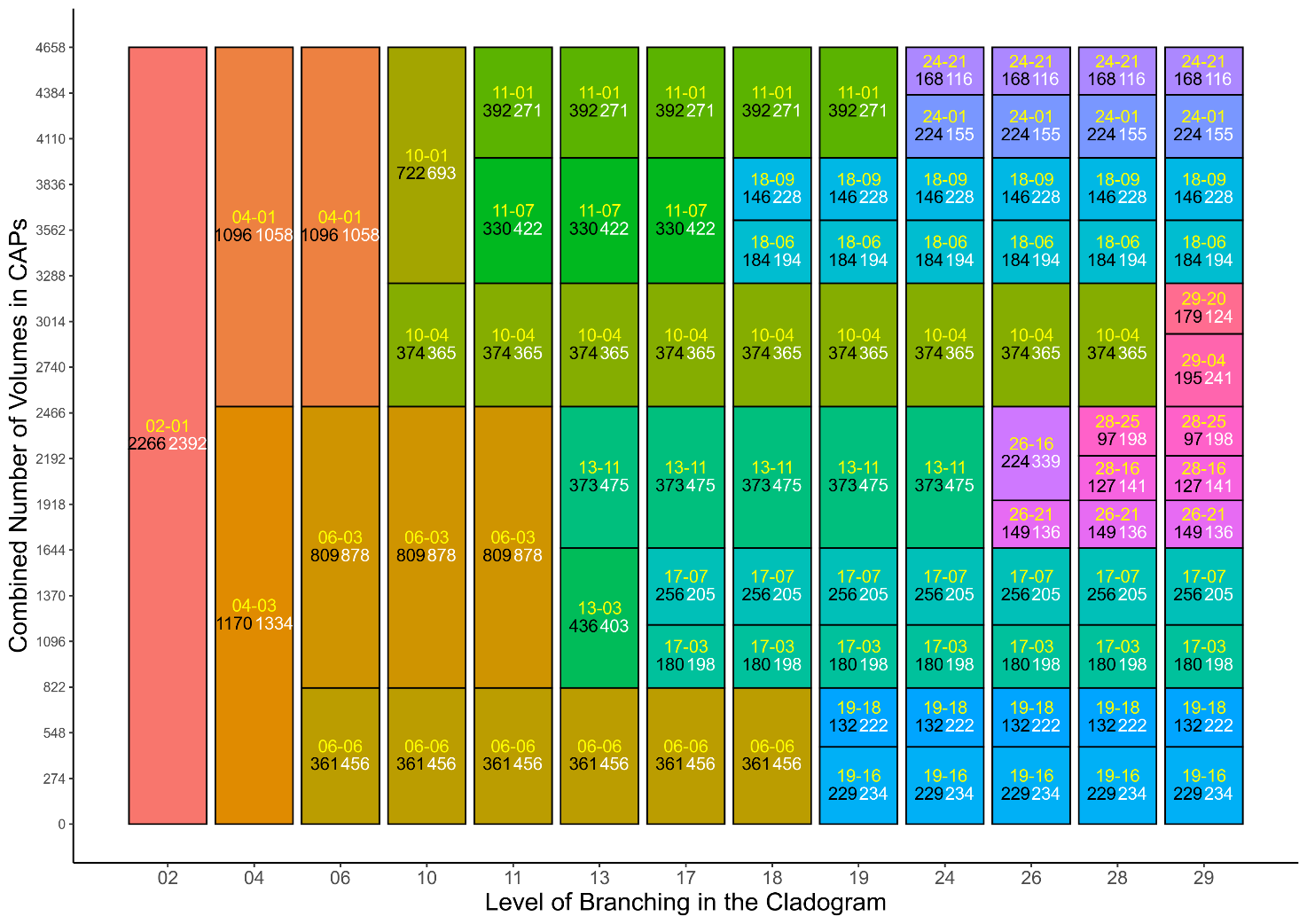


**Figure S1b.**

**Figures S1a-b. Proportions of ASD and TD participants' fMRI volumes in CAPs.** *The figures visualize the proportions of resting-state fMRI volumes in the CAPs at each branching level in the upper ("DMN-positive") and lower ("task-positive") halves of the cladogram of Figure 2. (Font colors: Yellow = CAP, black = ASD, white = TD)*


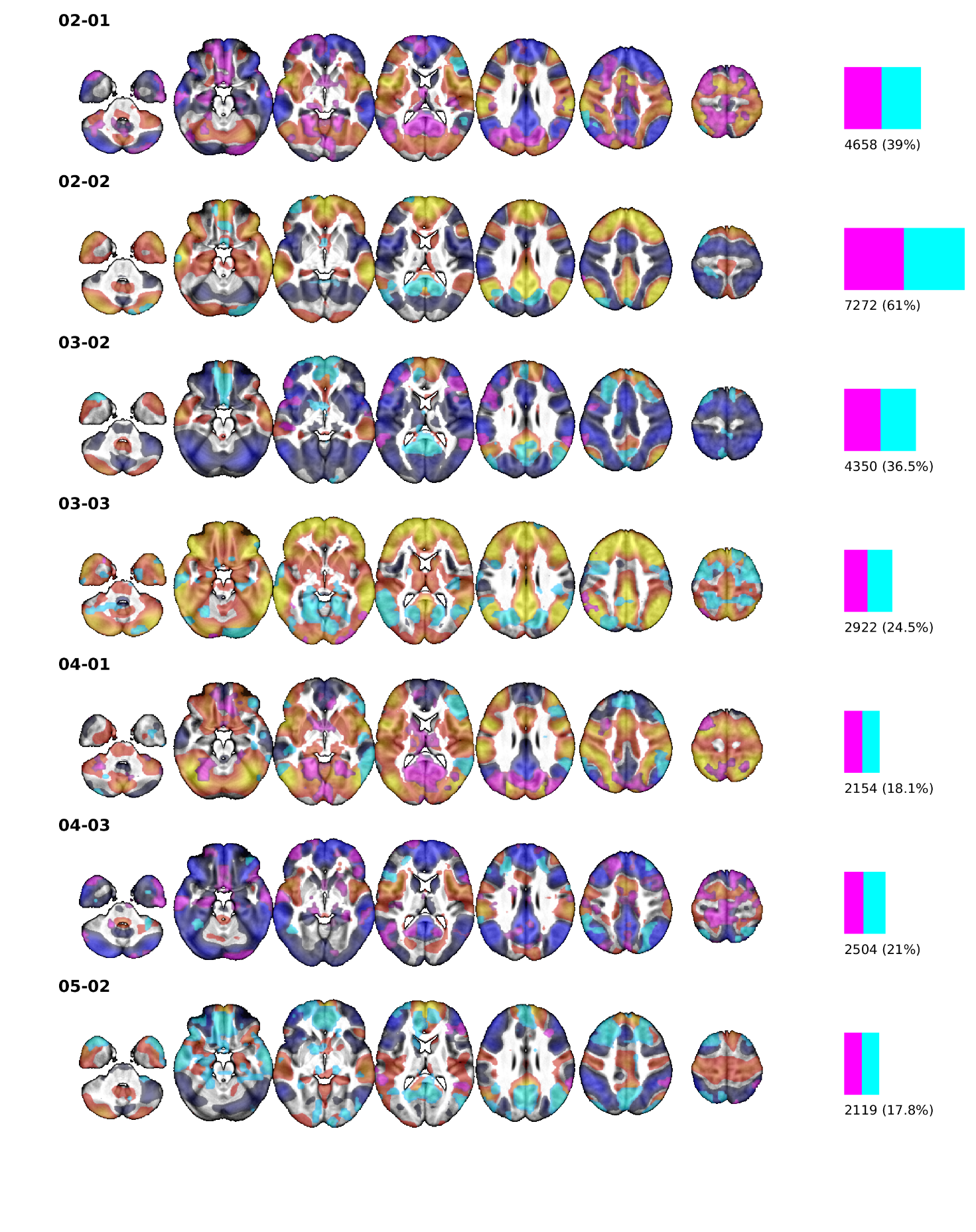
**Figure S2a.**


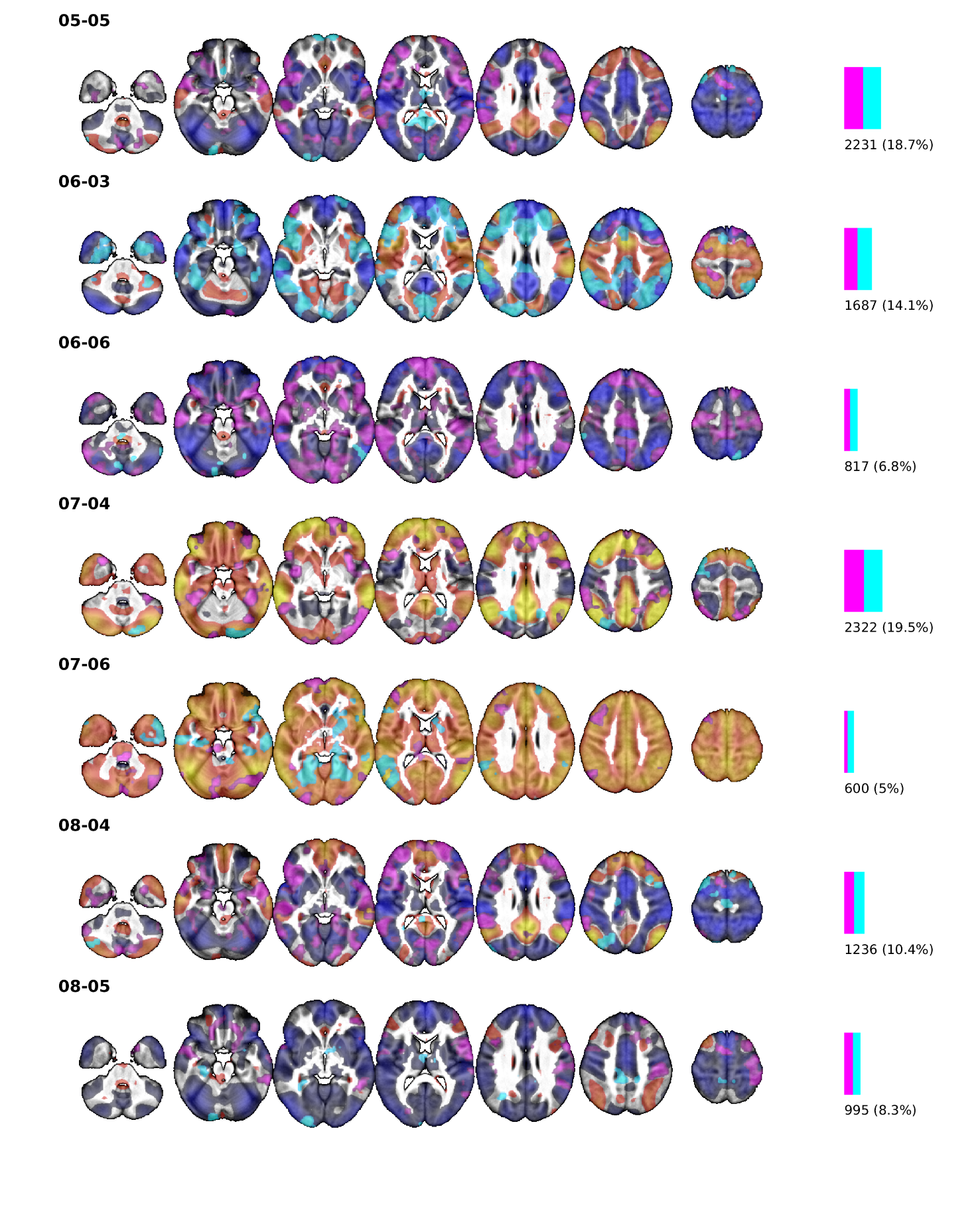
**Figure S2b.**


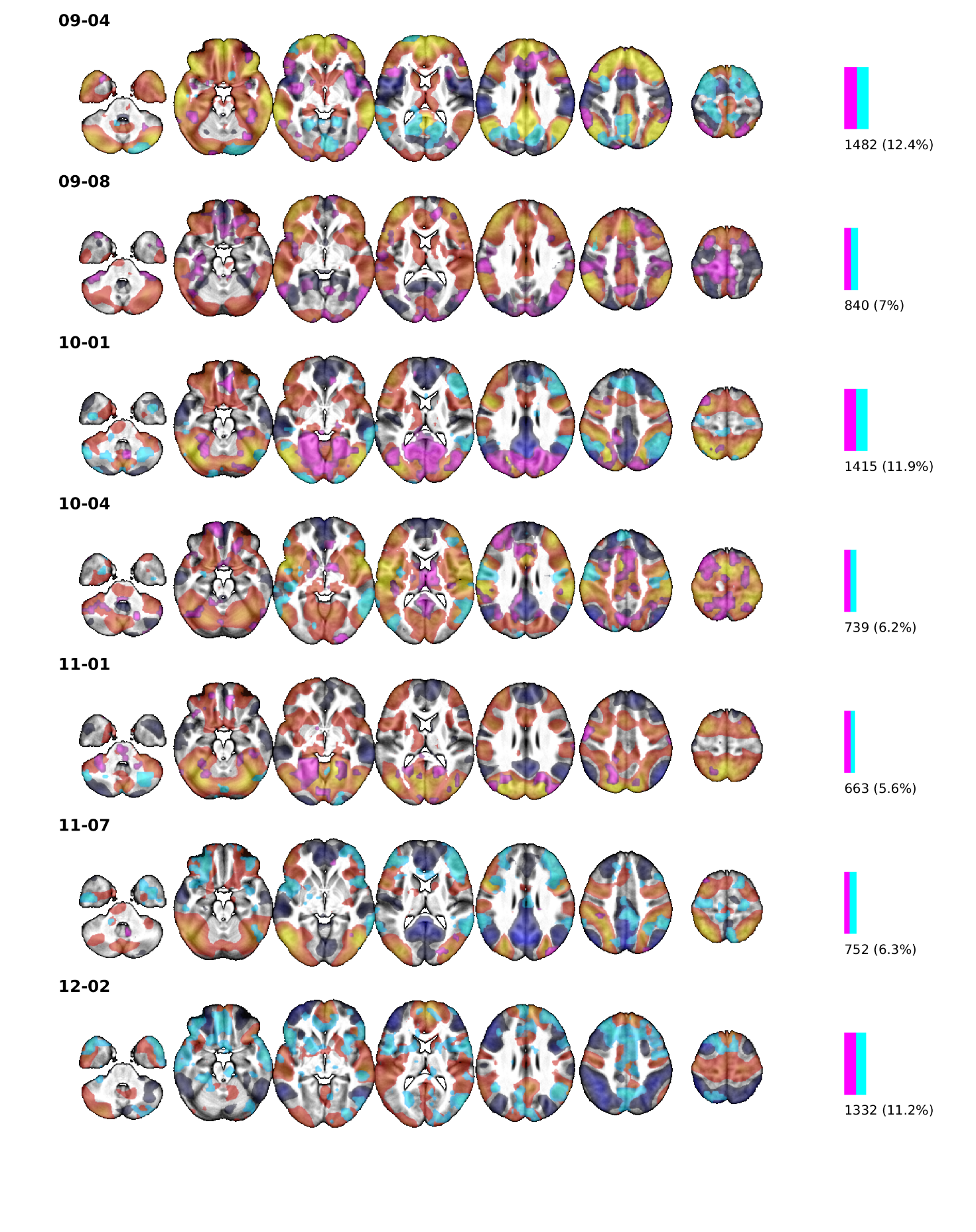
**Figure S2c.**


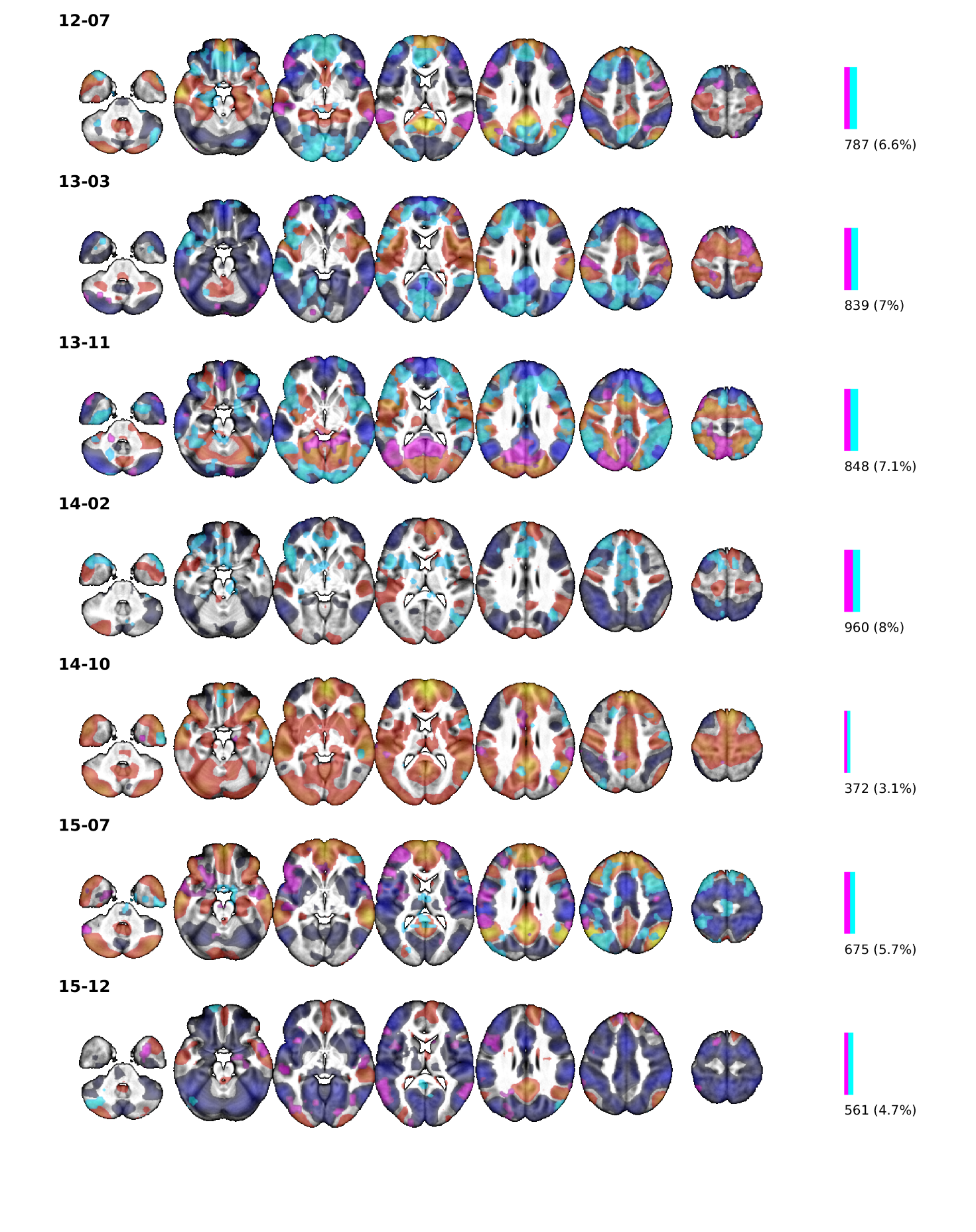
**Figure S2d.**


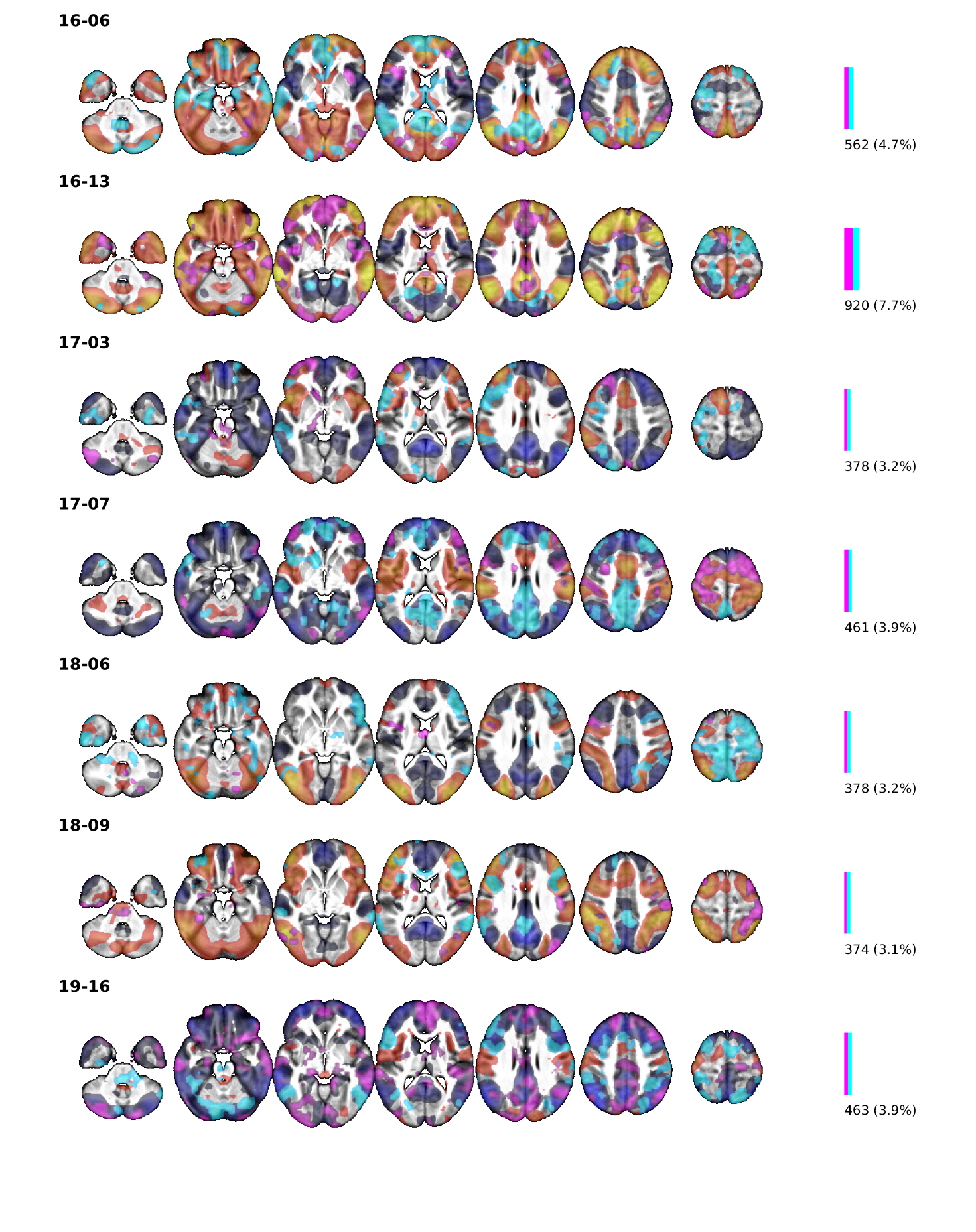
**Figure S2e.**


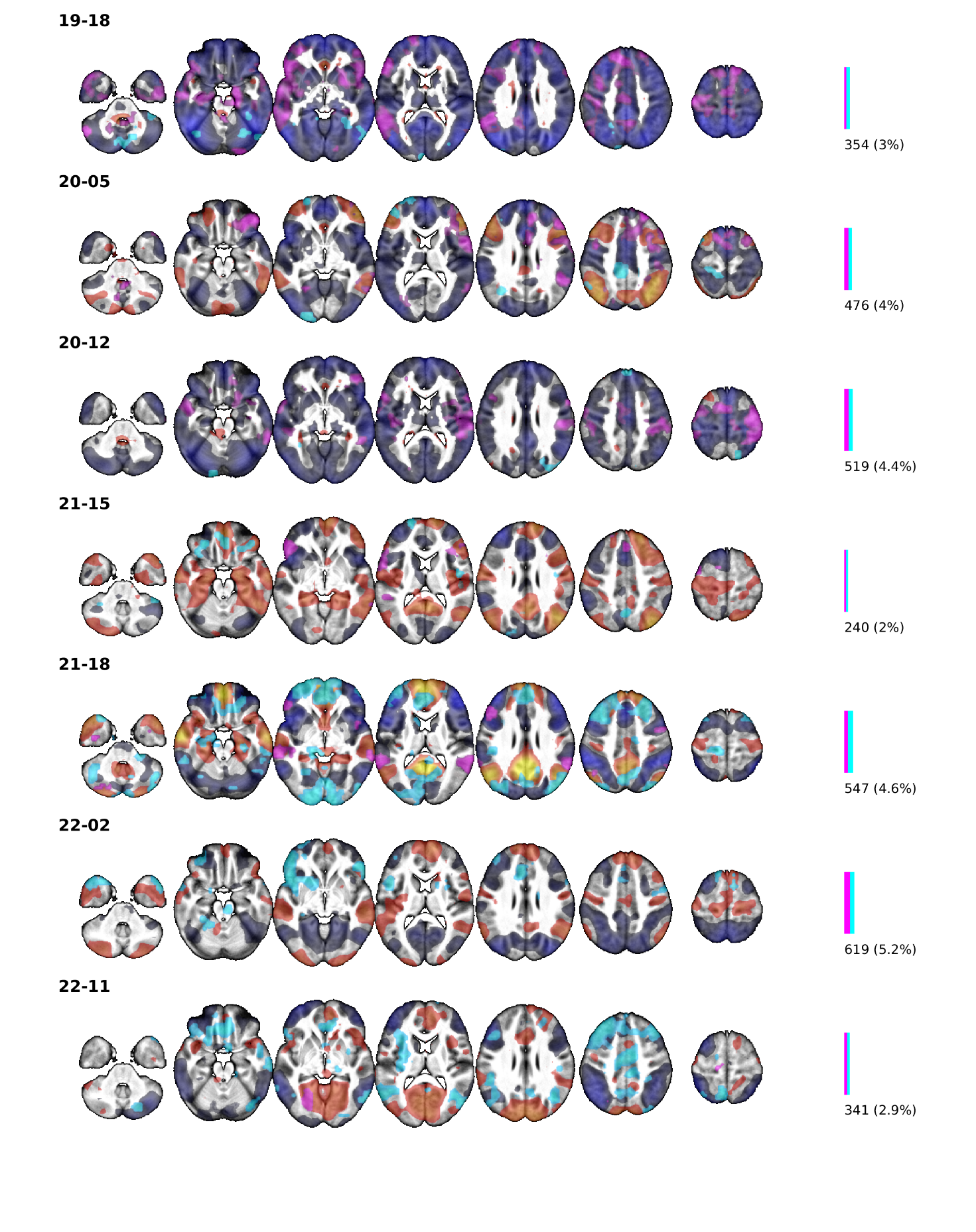
**Figure S2f.**


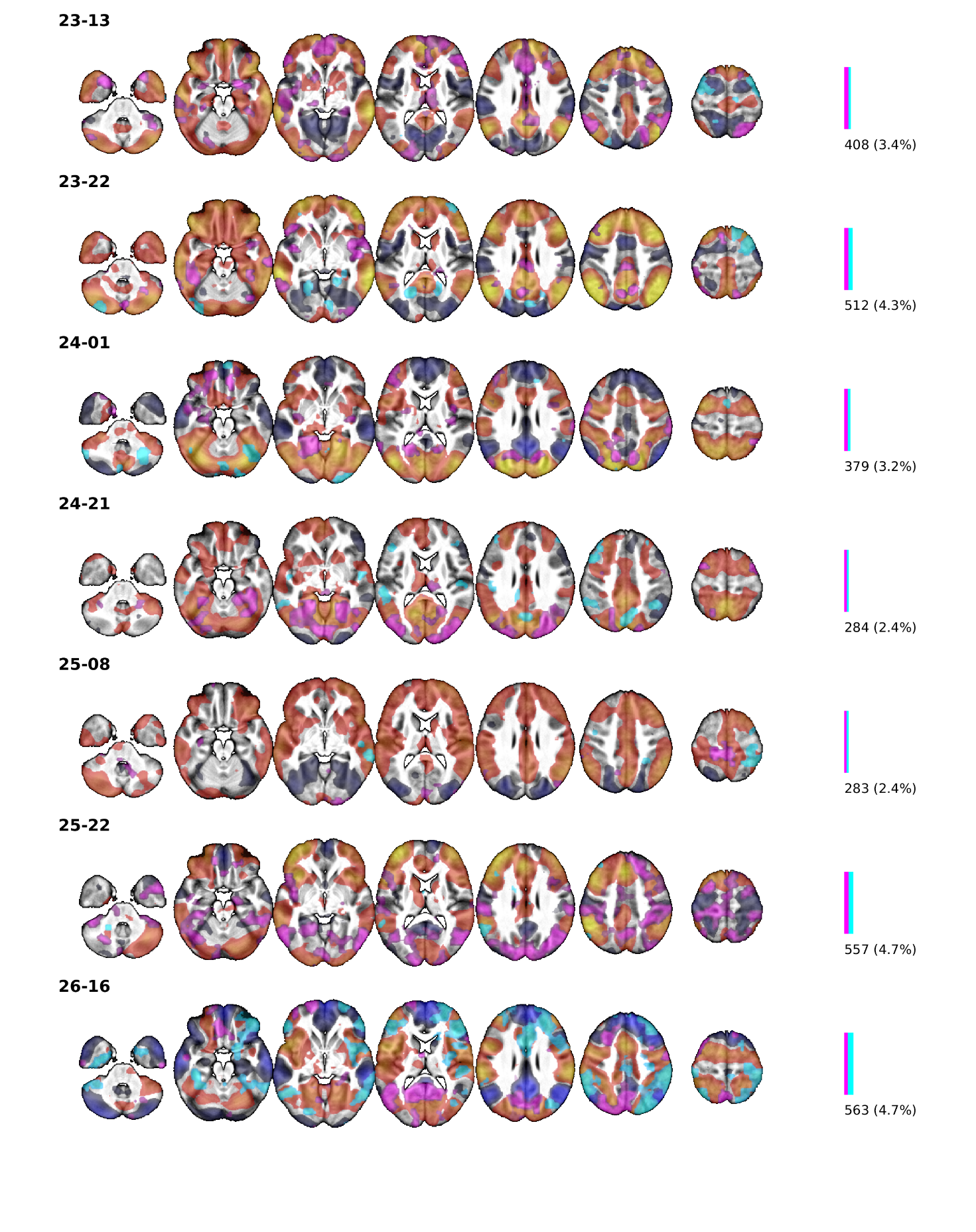
**Figure S2g.**


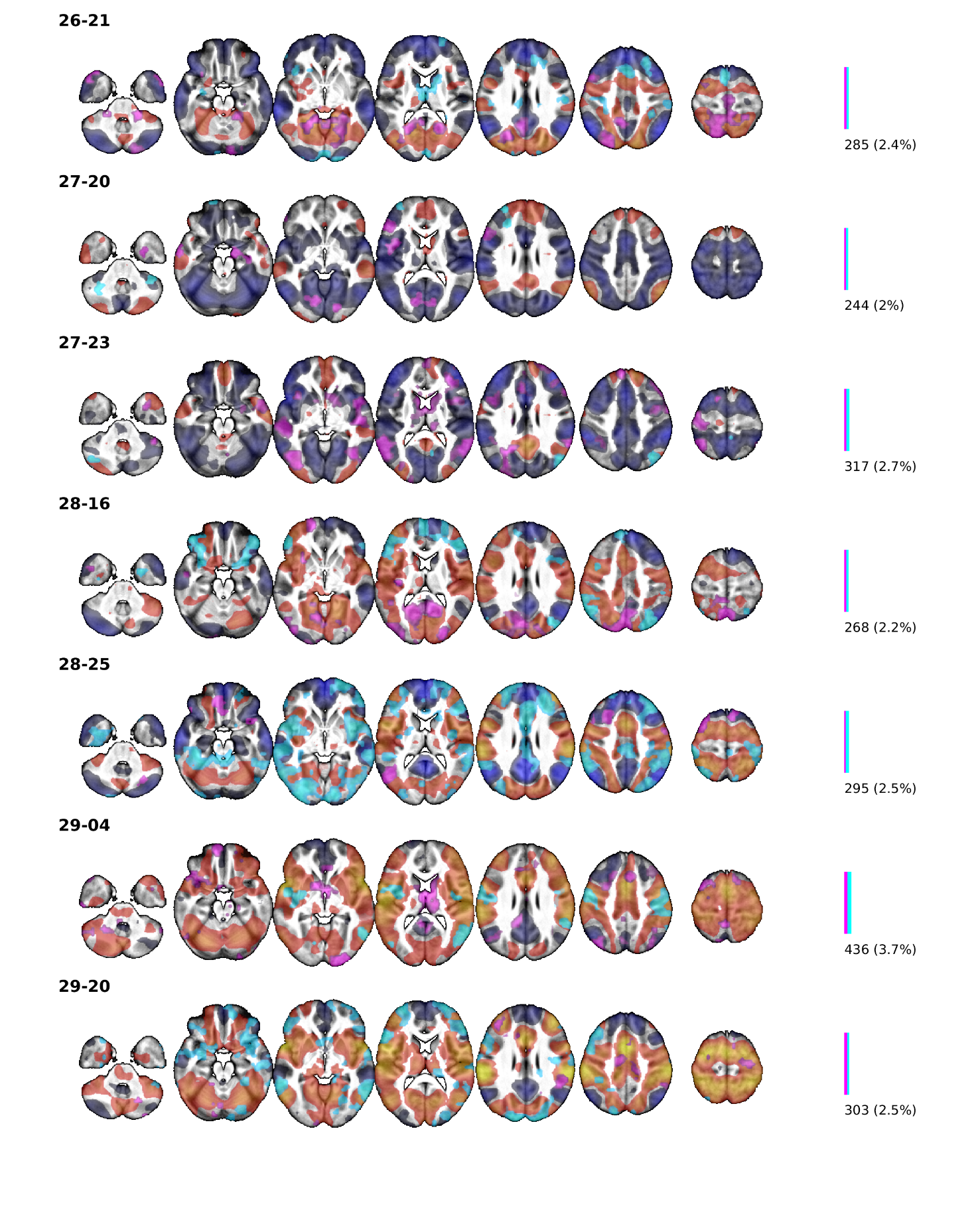
**Figure S2h.**


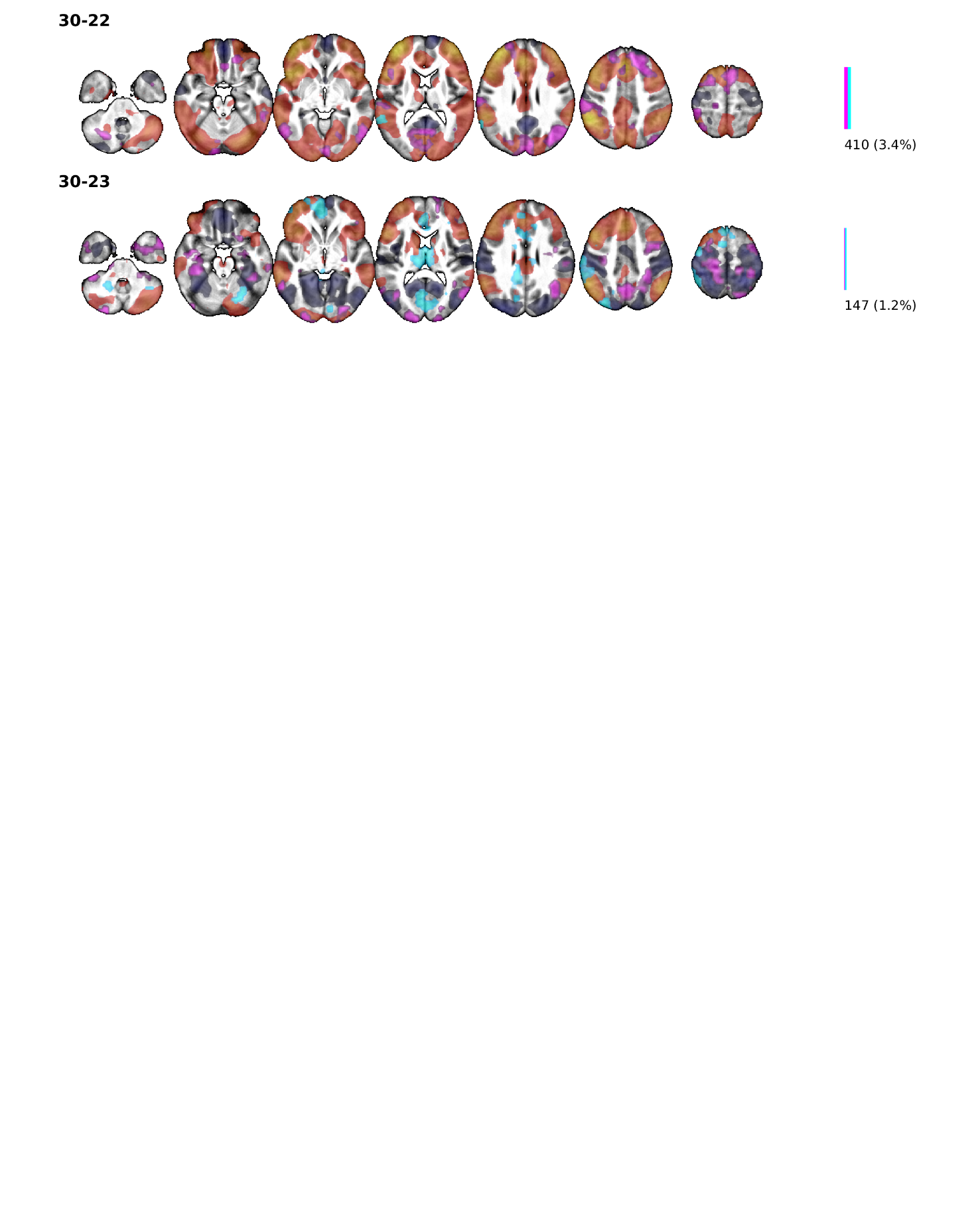
**Figure S2i.**

**Figures S2a-i. Study-specific anatomical template overlaid with CAPs' z-statistic maps and FDR corrected group difference maps, including CAP volume proportions compared to the whole data.** *Each CAP's z-statistic map slices are shown at the same levels. The z-statistic color keys range from -20 to -1.65 (blue) and from 1.65 to 20 (red-yellow). The range was selected to ensure that the small differences between some CAPs not noticeable in the heatmap of Figure 4 would be easier to detect here. The magenta clusters show brain areas in which the ASD group had significantly greater voxel-wise z-score values than the TD group. The cyan clusters show brain areas in which the TD group had significantly greater voxel-wise z-score values than the ASD group.*

*The fMRI volume count forming each CAP is shown in the right-hand column, followed by the percentual value compared to the whole fMRI data of 11 930 volumes. Each bar plot's relative length repeats the same information, and the magenta color depicts the proportion of the ASD group's and cyan TD group's volumes.*

**REFERENCES**

Anderson, M. J., and Robinson, J. (2001). Permutation Tests for Linear Models. *Aust. N. Z. J. Stat.* 43, 75–88. doi:10.1111/1467-842X.00156.

Andersson, J., Jenkinson, M., and Smith, S. (2007). Non-Linear Registration aka Spatial Normalisation FMRIB Technical Report TR07JA2. *https://www.fmrib.ox.ac.uk/datasets/techrep/*.

Aurich, N. K., Alves Filho, J. O., Marques da Silva, A. M., and Franco, A. R. (2015). Evaluating the reliability of different preprocessing steps to estimate graph theoretical measures in resting state fMRI data. *Front. Neurosci.* 9. doi:10.3389/fnins.2015.00048.

Bengtsson, H. (2016). *R.matlab: Read and Write MAT Files and Call MATLAB from Within R*. Available at: https://CRAN.R-project.org/package=R.matlab [Accessed August 11, 2019].

Benjamini, Y., and Hochberg, Y. (1995). Controlling the False Discovery Rate: A Practical and Powerful Approach to Multiple Testing. *J. R. Stat. Soc. Ser. B Methodol.* 57, 289–300.

Castellazzi, G., Palesi, F., Casali, S., Vitali, P., Sinforiani, E., Wheeler-Kingshott, C. A. M., et al. (2014). A comprehensive assessment of resting state networks: bidirectional modification of functional integrity in cerebro-cerebellar networks in dementia. *Front. Neurosci.* 8. doi:10.3389/fnins.2014.00223.

Chen, Z., and Calhoun, V. (2018). Effect of Spatial Smoothing on Task fMRI ICA and Functional Connectivity. *Front. Neurosci.* 12. doi:10.3389/fnins.2018.00015.

Cox, R. W. (1996). AFNI: software for analysis and visualization of functional magnetic resonance neuroimages. *Comput. Biomed. Res. Int. J.* 29, 162–173. doi:10.1006/cbmr.1996.0014.

Cox, R. W., and Hyde, J. S. (1997). Software tools for analysis and visualization of fMRI data. *NMR Biomed.* 10, 171–178. doi:10.1002/(sici)1099-1492(199706/08)10:4/5<171::aid-nbm453>3.0.co;2-l.

Douaud, G., Smith, S., Jenkinson, M., Behrens, T., Johansen-Berg, H., Vickers, J., et al. (2007). Anatomically related grey and white matter abnormalities in adolescent-onset schizophrenia. *Brain* 130, 2375–2386. doi:10.1093/brain/awm184.

Fox, M. D., Zhang, D., Snyder, A. Z., and Raichle, M. E. (2009). The Global Signal and Observed Anticorrelated Resting State Brain Networks. *J. Neurophysiol.* 101, 3270–3283. doi:10.1152/jn.90777.2008.

Galili, T. (2015). dendextend: an R package for visualizing, adjusting and comparing trees of hierarchical clustering. *Bioinformatics* 31, 3718–3720. doi:10.1093/bioinformatics/btv428.

Genovese, C. R., Lazar, N. A., and Nichols, T. (2002). Thresholding of statistical maps in functional neuroimaging using the false discovery rate. *NeuroImage* 15, 870–878. doi:10.1006/nimg.2001.1037.

Gold, S., Christian, B., Arndt, S., Zeien, G., Cizadlo, T., Johnson, D. L., et al. (1998). Functional MRI statistical software packages: a comparative analysis. *Hum. Brain Mapp.* 6, 73–84.

Good, C. D., Johnsrude, I. S., Ashburner, J., Henson, R. N. A., Friston, K. J., and Frackowiak, R. S. J. (2001). A Voxel-Based Morphometric Study of Ageing in 465 Normal Adult Human Brains. *NeuroImage* 14, 21–36. doi:10.1006/nimg.2001.0786.

Gotts, S. J., Saad, Z. S., Jo, H. J., Wallace, G. L., Cox, R. W., and Martin, A. (2013). The perils of global signal regression for group comparisons: a case study of Autism Spectrum Disorders. *Front. Hum. Neurosci.* 7. doi:10.3389/fnhum.2013.00356.

Jo, H. J., Gotts, S. J., Reynolds, R. C., Bandettini, P. A., Martin, A., Cox, R. W., et al. (2013). Effective Preprocessing Procedures Virtually Eliminate Distance-Dependent Motion Artifacts in Resting State FMRI. *J. Appl. Math.* 2013, 1–9. doi:10.1155/2013/935154.

Jo, H. J., Saad, Z. S., Simmons, W. K., Milbury, L. A., and Cox, R. W. (2010). Mapping sources of correlation in resting state FMRI, with artifact detection and removal. *NeuroImage* 52, 571–582. doi:10.1016/j.neuroimage.2010.04.246.

Liu, X., Chang, C., and Duyn, J. H. (2013). Decomposition of spontaneous brain activity into distinct fMRI co-activation patterns. *Front. Syst. Neurosci.* 7. doi:10.3389/fnsys.2013.00101.

Liu, X., Zhang, N., Chang, C., and Duyn, J. H. (2018). Co-activation patterns in resting-state fMRI signals. *NeuroImage* 180, 485–494. doi:10.1016/j.neuroimage.2018.01.041.

Manning, C. D., Raghavan, P., and Schütze, H. (2008). *Introduction to information retrieval*. New York: Cambridge University Press.

Müllner, D. (2013). fastcluster: Fast Hierarchical, Agglomerative Clustering Routines for R and Python. *J. Stat. Softw.* 53, 1–18.

Murphy, K., and Fox, M. D. (2017). Towards a consensus regarding global signal regression for resting state functional connectivity MRI. *NeuroImage* 154, 169–173. doi:10.1016/j.neuroimage.2016.11.052.

Murtagh, F., and Contreras, P. (2011). Methods of hierarchical clustering. *ArXiv Prepr. ArXiv11050121*. Available at: http://arxiv.org/abs/1105.0121 [Accessed July 18, 2015].

Neuwirth, E. (2014). *RColorBrewer: ColorBrewer Palettes*. Available at: https://CRAN.R-project.org/package=RColorBrewer.

Nichols, T. (2013). Notes on creating a standardized version of DVARS. Available at: http://citeseerx.ist.psu.edu/viewdoc/download?doi=10.1.1.411.9059&rep=rep1&type=pdf [Accessed May 17, 2016].

Paradis, E., and Schliep, K. (2018). ape 5.0: an environment for modern phylogenetics and evolutionary analyses in R. *Bioinformatics* 35, 526–528.

Patenaude, B., Smith, S. M., Kennedy, D. N., and Jenkinson, M. (2011). A Bayesian model of shape and appearance for subcortical brain segmentation. *NeuroImage* 56, 907–922. doi:10.1016/j.neuroimage.2011.02.046.

Power, J. D., Mitra, A., Laumann, T. O., Snyder, A. Z., Schlaggar, B. L., and Petersen, S. E. (2014). Methods to detect, characterize, and remove motion artifact in resting state fMRI. *NeuroImage* 84, 320–341. doi:10.1016/j.neuroimage.2013.08.048.

Power, J. D., Schlaggar, B. L., and Petersen, S. E. (2015). Recent progress and outstanding issues in motion correction in resting state fMRI. *NeuroImage* 105, 536–551. doi:10.1016/j.neuroimage.2014.10.044.

Pruim, R. H. R., Mennes, M., Buitelaar, J. K., and Beckmann, C. F. (2015a). Evaluation of ICA-AROMA and alternative strategies for motion artifact removal in resting state fMRI. *NeuroImage* 112, 278–287. doi:10.1016/j.neuroimage.2015.02.063.

Pruim, R. H. R., Mennes, M., van Rooij, D., Llera, A., Buitelaar, J. K., and Beckmann, C. F. (2015b). ICA-AROMA: A robust ICA-based strategy for removing motion artifacts from fMRI data. *NeuroImage* 112, 267–277. doi:10.1016/j.neuroimage.2015.02.064.

R Core Team (2017). *R: A Language and Environment for Statistical Computing*. Vienna, Austria: R Foundation for Statistical Computing Available at: https://www.R-project.org/.

Rorden, C., and Brett, M. (2000). Stereotaxic display of brain lesions. *Behav. Neurol.* 12, 191–200. doi:10.1155/2000/421719.

Sakai, R., Winand, R., Verbeiren, T., Moere, A. V., and Aerts, J. (2014). dendsort: modular leaf ordering methods for dendrogram representations in R. *F1000Research* 3. doi:10.12688/f1000research.4784.1.

Shen, J. (2014). Tools for NIfTI and ANALYZE image - File Exchange - MATLAB Central. *Httpsemathworkscommatlabcentralfileexchange8797-Tools--Nifti--Anal.-Image*. Available at: http://se.mathworks.com/matlabcentral/fileexchange/8797-tools-for-nifti-and-analyze-image [Accessed January 1, 2018].

Singhal, A. (2001). Modern information retrieval: A brief overview. *IEEE Data Eng Bull* 24, 35–43.

Smith, S. M., Fox, P. T., Miller, K. L., Glahn, D. C., Fox, P. M., Mackay, C. E., et al. (2009). Correspondence of the brain's functional architecture during activation and rest. *Proc. Natl. Acad. Sci.* 106, 13040–13045.

Smith, S. M., Jenkinson, M., Woolrich, M. W., Beckmann, C. F., Behrens, T. E. J., Johansen-Berg, H., et al. (2004). Advances in functional and structural MR image analysis and implementation as FSL. *NeuroImage* 23, S208–S219. doi:10.1016/j.neuroimage.2004.07.051.

Starck, T., Nikkinen, J., Rahko, J., Remes, J., Hurtig, T., Haapsamo, H., et al. (2013). Resting state fMRI reveals a default mode dissociation between retrosplenial and medial prefrontal subnetworks in ASD despite motion scrubbing. *Front. Hum. Neurosci.* 7. doi:10.3389/fnhum.2013.00802.

Thornburgh, C. L., Narayana, S., Rezaie, R., Bydlinski, B. N., Tylavsky, F. A., Papanicolaou, A. C., et al. (2017). Concordance of the Resting State Networks in Typically Developing, 6-to 7-Year-Old Children and Healthy Adults. *Front. Hum. Neurosci.* 11. doi:10.3389/fnhum.2017.00199.

Uddin, L. Q., Supekar, K., Lynch, C. J., Cheng, K. M., Odriozola, P., Barth, M. E., et al. (2015). Brain State Differentiation and Behavioral Inflexibility in Autism. *Cereb. Cortex* 25, 4740–4747. doi:10.1093/cercor/bhu161.

Vossel, S., Geng, J. J., and Fink, G. R. (2014). Dorsal and Ventral Attention Systems. *The Neuroscientist* 20, 150–159. doi:10.1177/1073858413494269.

Warnes, G. R., Bolker, B., Bonebakker, L., Gentleman, R., Liaw, W. H. A., Lumley, T., et al. (2020). *gplots: Various R Programming Tools for Plotting Data*. Available at: https://CRAN.R-project.org/package=gplots.

White, T., Muetzel, R., Schmidt, M., Langeslag, S. J. E., Jaddoe, V., Hofman, A., et al. (2014). Time of acquisition and network stability in pediatric resting-state functional magnetic resonance imaging. *Brain Connect.* 4, 417–427. doi:10.1089/brain.2013.0195.

Wickham, H. (2011). The Split-Apply-Combine Strategy for Data Analysis. *J. Stat. Softw.* 40, 1–29. doi:10.18637/jss.v040.i01.

Wickham, H. (2016). *ggplot2: Elegant Graphics for Data Analysis*. Springer-Verlag New York Available at: https://ggplot2.tidyverse.org.

Wickham, H., François, R., Henry, L., and Müller, K. (2020). *dplyr: A Grammar of Data Manipulation*. Available at: https://CRAN.R-project.org/package=dplyr.

Winkler, A. M., Ridgway, G. R., Webster, M. A., Smith, S. M., and Nichols, T. E. (2014). Permutation inference for the general linear model. *NeuroImage* 92, 381–397. doi:10.1016/j.neuroimage.2014.01.060.

Yeo, B. T. T., Krienen, F. M., Sepulcre, J., Sabuncu, M. R., Lashkari, D., Hollinshead, M., et al. (2011). The organization of the human cerebral cortex estimated by intrinsic functional connectivity. *J. Neurophysiol.* 106, 1125–1165. doi:10.1152/jn.00338.2011.

Yeo, B. T. T., Tandi, J., and Chee, M. W. L. (2015). Functional connectivity during rested wakefulness predicts vulnerability to sleep deprivation. *NeuroImage* 111, 147–158. doi:10.1016/j.neuroimage.2015.02.018.

Yu, G., Smith, D. K., Zhu, H., Guan, Y., and Lam, T. T.-Y. (2017). ggtree : an r package for visualization and annotation of phylogenetic trees with their covariates and other associated data. *Methods Ecol. Evol.* 8, 28–36. doi:10.1111/2041-210X.12628.
